# The Tax-Inducible Actin-Bundling Protein Fascin Is Crucial for Release and Cell-to-Cell Transmission of Human T-Cell Leukemia Virus Type 1 (HTLV-1)

**DOI:** 10.1371/journal.ppat.1005916

**Published:** 2016-10-24

**Authors:** Christine Gross, Veit Wiesmann, Sebastian Millen, Martina Kalmer, Thomas Wittenberg, Jan Gettemans, Andrea K. Thoma-Kress

**Affiliations:** 1 Institute of Clinical and Molecular Virology, Friedrich-Alexander-Universität Erlangen-Nürnberg (FAU), Erlangen, Germany; 2 Fraunhofer Institute for Integrated Circuits IIS, Erlangen, Germany; 3 Department of Biochemistry, Faculty of Medicine and Health Sciences, Campus Rommelaere, Ghent University, Ghent, Belgium; University of Wisconsin, UNITED STATES

## Abstract

The delta-retrovirus Human T-cell leukemia virus type 1 (HTLV-1) preferentially infects CD4^+^ T-cells via cell-to-cell transmission. Viruses are transmitted by polarized budding and by transfer of viral biofilms at the virological synapse (VS). Formation of the VS requires the viral Tax protein and polarization of the host cytoskeleton, however, molecular mechanisms of HTLV-1 cell-to-cell transmission remain incompletely understood. Recently, we could show Tax-dependent upregulation of the actin-bundling protein Fascin (FSCN-1) in HTLV-1-infected T-cells. Here, we report that Fascin contributes to HTLV-1 transmission. Using single-cycle replication-dependent HTLV-1 reporter vectors, we found that repression of endogenous Fascin by short hairpin RNAs and by Fascin-specific nanobodies impaired gag p19 release and cell-to-cell transmission in 293T cells. In Jurkat T-cells, Tax-induced Fascin expression enhanced virus release and Fascin-dependently augmented cell-to-cell transmission to Raji/CD4^+^ B-cells. Repression of Fascin in HTLV-1-infected T-cells diminished virus release and gag p19 transfer to co-cultured T-cells. Spotting the mechanism, flow cytometry and automatic image analysis showed that Tax-induced T-cell conjugate formation occurred Fascin-independently. However, adhesion of HTLV-1-infected MT-2 cells in co-culture with Jurkat T-cells was reduced upon knockdown of Fascin, suggesting that Fascin contributes to dissemination of infected T-cells. Imaging of chronically infected MS-9 T-cells in co-culture with Jurkat T-cells revealed that Fascin’s localization at tight cell-cell contacts is accompanied by gag polarization suggesting that Fascin directly affects the distribution of gag to budding sites, and therefore, indirectly viral transmission. In detail, we found gag clusters that are interspersed with Fascin clusters, suggesting that Fascin makes room for gag in viral biofilms. Moreover, we observed short, Fascin-containing membrane extensions surrounding gag clusters and clutching uninfected T-cells. Finally, we detected Fascin and gag in long-distance cellular protrusions. Taken together, we show for the first time that HTLV-1 usurps the host cell factor Fascin to foster virus release and cell-to-cell transmission.

## Author Summary

Human T-cell leukemia virus type 1 (HTLV-1) is the only human retrovirus causing cancer and is transmitted via breast feeding, sexual intercourse, and cell-containing blood products. Efficient infection of CD4^+^ T-cells occurs via polarized budding of virions or via cell surface transfer of viral biofilms at a tight, specialized cell-cell contact, the virological synapse (VS). The viral protein Tax and polarization of the host cell cytoskeleton are crucial for formation of the VS, however, only little is known about the link between Tax and remodeling of the cytoskeleton to foster viral spread. The actin-bundling protein Fascin has evolved as a therapeutic target in several types of cancer. Here, we show that Fascin is also crucial for release and transmission of the tumorvirus HTLV-1. Since Fascin is a transcriptional target gene of Tax in T-cells, our work provides a link between Tax’s activity and virus transmission. Visualization of cell-cell contacts between infected and uninfected T-cells suggests a role of Fascin in viral transmission potentially by facilitating the transport of viral proteins to budding sites. Thus, Fascin is not only crucial for metastasis of tumors, but also for transmission of HTLV-1 and is a new cellular target to counteract HTLV-1.

## Introduction

Human T-cell leukemia virus type 1 (HTLV-1), which infects approximately 5–10 million people worldwide [1], is the only human retrovirus causing cancer: adult T-cell leukemia/lymphoma (ATL), a fatal neoplasia of CD4^+^ T-cells [2–4]. Further, HTLV-1 is the causative agent of a neurodegenerative, inflammatory disease, HTLV-1-associated myelopathy/tropical spastic paraparesis (HAM/TSP) [5,6]. Both diseases can develop as a consequence of prolonged viral persistence in T-cells after a clinical latency of decades in 1–5% (ATL) or 3–5% (HAM/TSP) of infected individuals [7,8].

Activated CD4^+^ T-cells are the main and preferential target for HTLV-1 infection, but the virus is also present in very low amounts in other cell types including CD8^+^ T-cells, monocytes, and dendritic cells (DC) [9]. After binding to its receptor, which is composed of the glucose transporter GLUT-1, neuropilin-1 (NRP-1) and heparan sulfate proteoglycans (HSPGs) [10–12], HTLV-1 integrates into the host cell genome. The virus is mainly maintained in its provirus form (9.1 kb), which is flanked by long terminal repeats (LTR) in both the 5’ and 3’ region. In addition to structural proteins and enzymes common for retroviruses, HTLV-1 encodes regulatory (Tax, Rex) and accessory (p12/p8, p13, p30, HBZ) proteins [13]. HTLV-1 replicates either by infecting new cells or by mitotic division and clonal expansion of infected T-cells [14–16].

Efficient infection of CD4^+^ T-cells requires cell-cell contacts and coordinated steps of the virus infectious cycle with events in the cell-cell adhesion process. Thus, transmission of HTLV-1 occurs via breast feeding, sexual intercourse, and cell-containing blood products [9,17]. Unlike human immunodeficiency virus (HIV) or murine leukemia virus (MLV), cell-free transmission of HTLV-1 to T-cells is inefficient. Therefore, only a limited amount of poorly infectious viral particles is produced from infected lymphocytes and free virions can hardly be detected in infected individuals [18–20]. Thus far, two types of cell-cell contacts have been described to be critical for HTLV-1 transmission, tight cell-cell contacts and cellular conduits [21,22]. For transmission at tight cell-cell contacts, two non-exclusive mechanisms of virus transmission at the virological synapse (VS), a virus-induced specialized cell-cell contact [23], have been proposed [17]: (1) polarized budding of HTLV-1 into synaptic clefts [21], and (2) cell surface transfer of viral biofilms [24]. The latter consist of extracellular, concentrated viral assemblies that are surrounded by components of the extracellular matrix and cellular lectins [24]. Beyond, transmission via biofilms seems to be a major route of transmission since removal of biofilms by heparin treatment impairs cell-to-cell transmission by 80% *in vitro* [24]. Independent of the route of HTLV-1 transmission, viral particles are thought to be transmitted in confined areas protected from the immune response of the host *in vivo*. Moreover, cytoskeletal remodeling and cell-cell contacts are a prerequisite for all routes of virus transmission [21,25]. Although it is known that the viral protein Tax and polarization of the host cell cytoskeleton are crucial for formation of the VS and for HTLV-1 transmission (for details see: [17,23]), only little is known about the link between Tax and remodeling of the cytoskeleton to foster viral spread.

The regulatory protein Tax is essential for viral replication due to strong enhancement of viral mRNA synthesis by transactivating the HTLV-1 LTR (U3R) promoter. Beyond, Tax is a potent transactivator of cellular transcription and important for initiating oncogenic transformation [13]. Tax is also critical for HTLV-1 transmission since Tax cooperates with intercellular adhesion molecule 1 (ICAM-1), thereby inducing polarization of the microtubule organizing center (MTOC) at the VS [26] and thus, enhancing HTLV-1 cell-to-cell transfer. Furthermore, Tax enhances both actin- and tubulin-dependent transmission of virus-like particles (VLPs; [25]). However, only few host cell factors with a role in Tax-induced virus transmission have been characterized. Among those is ICAM-1, which is induced by Tax and cooperates with Tax in VS formation [26,27]. The Tax-induced small GTP-binding protein GEM enhances cellular migration, conjugate formation, and thus, is required for viral transmission [28].

In our search of novel target genes of Tax with a putative role in virus transmission, we have previously identified the evolutionary conserved actin-bundling protein and tumor marker Fascin as a new host cell factor strongly induced by Tax [29]. Fascin cross-links filamentous actin and stabilizes cellular protrusions, filopodia, and invadopodia [30]. Recent work shows that Fascin also interacts with microtubules to regulate adhesion dynamics and cell migration [31]. Fascin has evolved as a therapeutic target in several types of cancer since Fascin expression is associated with metastasis in malignant tumors and it correlates with clinical aggressiveness of some tumors [30]. In hematopoietic cells, Fascin is expressed in mature DC where it is important for stability of dendrites and for formation of the immunological synapse [32], while no expression of Fascin can be detected in unstimulated human T-cells [33]. We found that expression of Fascin is a common feature of chronically HTLV-1-infected T-cell lines. Fascin colocalizes with actin in the cytoplasm and at the membrane of HTLV-1-infected cells. Furthermore, knockdown of Fascin reduces the invasive capacity of HTLV-1-infected ATL-derived T-cells into extracellular matrix [29]. Since expression of Tax is sufficient to induce expression of Fascin [29,34] and Tax enhances actin-dependent virus transmission [25], we now asked whether Fascin affects HTLV-1 cell-to-cell transfer.

Here, we report that Fascin is crucial for release and cell-to-cell transmission of HTLV-1 in different cell model systems. While T-cell conjugate formation is Fascin-independent, cell adhesion of infected cells in co-culture with uninfected cells is impaired upon repression of Fascin. Imaging of Fascin and the viral gag protein at cell-cell contacts suggests a role of Fascin in transmission potentially by redirecting viral proteins to budding sites. Thus, Fascin as a major contributor to HTLV-1 transmission provides a link between Tax’s activity and virus transmission.

## Materials and Methods

### Cell culture

293T cells (kindly provided by Ralph Grassmann (deceased), FAU, Erlangen, Germany) were cultured in DMEM containing 10% fetal calf serum (FCS), L-glutamine (0.35g/l) and penicillin/streptomycin (Pen/Strep; 0.12g/l each). For selection of stable 293T cells carrying shRNAs, 4μg/ml puromycin was added to the media. The CD4^+^ T-cell line Jurkat (ATCC, LGC Standards GmbH, Wesel, Germany) from acute lymphoblastic leukemia was cultured in RPMI 1640M, Panserin, 10% FCS, L-glutamine and Pen/Strep [35]. The human Epstein-Barr virus (EBV)-positive B-cell line Raji derived from Burkitt’s lymphoma containing the surface receptor CD4 (Raji/CD4^+^) was a kind gift from Vineet N. Kewal Ramani (NIH, Frederick, Maryland, USA) and was cultured in RPMI 1640M, Panserin, 10% FCS, L-glutamine and Pen/Strep containing 500μg/ml geneticin to ensure retainment of the CD4 receptor [36]. The HTLV-1 *in vitro* transformed CD4^+^ T-cell line MT-2 [3] and the ATL-derived CD4^+^ T-cell line HuT-102 [2,37] were kindly provided by Ralph Grassmann (deceased, FAU, Erlangen, Germany) and were cultured in RPMI 1640M, 10% FCS and Pen/Strep. The HTLV-1 *in vitro* transformed T-cell line MS-9 (containing a single, full-length provirus) [38] was a kind gift from Charles Bangham (Imperial College, London, UK) and was cultured in RPMI 1640M, Panserin, 20% FCS, Pen/Strep and 100U/ml interleukin 2 (IL-2). All cell lines were checked for integrity by DNA profiling of eight different and highly polymorphic short tandem repeat loci (DSMZ, Braunschweig, Germany).

### Plasmids

#### Expression Plasmids

The following plasmids were used: the Tax-expression vector pEFneo-Tax1 (pEFTax) [39]; the respective control vector pEFneo (pEF); the control vector pcDNA3 (LifeTechnologies GmbH, Darmstadt, Germany); the Tax-expression vector pEGFPC1-Tax (GFP-Tax; [40]); the retroviral expression vector encoding an unspecific shRNA control pSIREN-RetroQ-IRES-EGFP-shNonsense (shNonsense; [41]) and the respective vectors containing shRNAs targeting Fascin (shFascin4, shFascin5; [29,42]); the expression plasmids encoding a control nanobody (MOMGFPNb (GFPNb)) or Fascin-specific nanobodies (MOMFASNb2 (FASNb2); MOMFASNb5 (FASNb5)) in pcDNA3.1/V5His/TOPO carrying a mitochondrial outer membrane (MOM) sequence to delocalize endogenous Fascin (MOM V5 pcDNA3.1; [43]).

#### Single-cycle replication-dependent reporter system

The single-cycle replication-dependent reporter system vectors used here were a kind gift from Gisela Heidecker-Fanning (NIH, Frederick, Maryland, USA) and have been described before [19,25]. Briefly, HTLV-1 reporter vectors contain an antisense-oriented reporter cassette composed of a CMV-driven *luciferase* (*luc*) gene that is interrupted by a sense-oriented γ-globin intron and followed by a TK poly(A) signal (pCRU5HT1inluc; inluc) [25]. After transcription, the intron is spliced via splice donor and splice acceptor sites. The antisense orientation of the *luc* gene precludes translation of *luc* mRNA in transfected cells. The *luc* reporter vector contains a packaging signal allowing incorporation of reporter RNA into viral particles, which are encoded by a co-transfected viral packaging plasmid that expresses all HTLV-1 gene products, including the wildtype envelope (env) of HTLV-1 (pCMVHT1M; wildtype; wt) [25]. In some experiments, packaging plasmids carrying a deletion of env (pCMVHT1-ΔEnv; Δenv) were used and those were pseudotyped with VSV-G (glycoprotein G of the Vesicular stomatitis virus). After infection of new cells, reporter mRNAs are reversely transcribed and *luc* activity can be measured.

### Transfections

In general, 10^7^ Jurkat T-cells were transiently transfected by electroporation using the Gene Pulser X Electroporation System (BioRad, Munich, Germany) at 290V and 1500μF. Cells were transfected using a total of 50 or 100μg of DNA. 5x10^5^ 293T cells or stable 293T cell lines that carry shNonsense, shFascin5 or shFascin4 were seeded in 6-well plates 24h prior to transfection. Cells were transfected with GeneJuice reagent (Merck Millipore, Darmstadt, Germany) according to the manufacturer’s protocol using a total amount of 2μg DNA.

### Generation of stable cell lines

#### Production of retroviral particles and transduction of HuT-102 and MT-2 cells with shRNAs

To produce retroviral particles, GP-293 cells (Clontech, Mountain View, CA, USA) were seeded in 10cm dishes and transfected with 10μg of the retroviral expression plasmid pSIREN-RetroQ-IRES-EGFP-shNonsense or -shFascin5 (shNonsense, shFascin5) and 5μg of VSV-G by lipofection using lipofectamine and Optimem (LifeTechnologies GmbH). Five hours post transfection, 5ml DMEM (20% FCS, glutamine) were added to the cells. Two days after transfection, supernatants containing viral particles were harvested, pooled, and enriched by ultracentrifugation (2h, 24.000rpm, 4°C). Viral particles were resuspended in 3ml of culture medium containing 2μg/ml polybrene (hexadimethrine bromide; Sigma, Taufkirchen, Germany) and used to infect 10^6^ HTLV-1-infected MT-2 cells or HuT-102 cells as described previously [29]. After 24h, polybrene was removed from the transduced cells. From day 2 after transduction, HTLV-1–transformed cells were selected with culture medium containing 0.5μg/ml puromycin (Invitrogen, Karlsruhe, Germany) for two weeks and monitored by flow cytometry and western blot. After 2 weeks, selected cells were frozen at -80°C to generate cell stocks.

#### 293T cells with knockdown of Fascin

293T cells (see Cell culture) were transfected with 2μg shNonsense (control) or one of two specific shRNAs targeting Fascin (shFascin4, shFascin5) using GeneJuice reagent. From 48h after transfection, cells were selected with culture medium containing 4μg/ml puromycin for six days, monitored by flow cytometry and western blot, and cell stocks were frozen at -80°C.

### Infection assays using single-cycle replication-dependent reporter vectors

#### Co-cultures of Jurkat T-cells and Raji/CD4^+^ B-cells

For cell-to-cell infection experiments, 10^7^ Jurkat T-cells were transfected (see Transfections) with 27.5μg of the reporter vector pCRU5HT1inluc (inluc) and with 17.5μg of the HTLV-1 packaging plasmids pCMVHT1M containing either HTLV-1 wildtype env (wt), or with pCMVHT1-ΔEnv (Δenv) containing a deletion in env. The latter was pseudotyped by co-transfection of 5μg VSV-G. Additionally, cells were co-transfected with 20μg shRNA expression plasmids (shNonsense, shFascin5 or shFascin4) and 30μg pEFTax or the control pEF. Total transfected DNA was adjusted to 100μg with pcDNA3. In experiments using nanobodies, Jurkat T-cells transfected with inluc reporter, wt packaging plasmids, and with pEFTax were co-transfected with the control nanobody (25μg GFPNb) or one of two different nanobodies targeting Fascin (FASNb2, FASNb5; 25μg each). At 24h after transfection, media of Jurkat T-cells were changed and cells were co-cultured with Raji/CD4^+^ B-cells (each 10^6^ cells) for 48h at 37°C. Thereafter, cells were harvested to detect virus release by gag p19 ELISA, cell-to-cell transmission by luciferase assay and protein expression by western blot analysis. At least four independent experiments each performed in triplicates were performed.

#### Co-cultures of sh239T cells and 293T cells

5x10^5^ stable sh293T cells that carry shNonsense, shFascin5 or shFascin4 (see Generation of stable cell lines) were seeded 24h before transfection (see Transfections) using 0.6μg of the reporter vector inluc, 0.4μg of the packaging plasmids wt or Δenv pseudotyped with 0.1μg of VSV-G. Cells were co-transfected with 0.9μg pEFTax or the control pEF. In experiments using nanobodies, normal 293T cells were co-transfected with inluc reporter and Δenv packaging plasmids, and with the control nanobody (0.5μg GFPNb) or one of two different nanobodies targeting Fascin (0.5μg FASNb2; or 0.25, 0.5 or 1μg of FASNb5). Total transfected DNA was adjusted to 2μg with pcDNA3. After 24h, media were changed, and an additional 24h later cells were harvested to perform gag p19 ELISA, western blot analysis and luciferase assay (cell-to-cell transmission). At least four independent experiments each performed in triplicates were conducted.

### Transactivation assays

HuT-102 cells stably transduced with shRNAs targeting Fascin (shFascin5) or a control (shNonsense) were co-cultured with Jurkat T-cells that had been transfected 24h earlier with the luciferase reporter plasmid pGL3-U3R-Luc carrying the *luc* gene under control of the HTLV-1 core promoter U3R [44], or with the control plasmid pGL3-Basic (Promega, Mannheim, Germany). After 48h of co-culture at 37°C, luciferase reporter gene assays were performed. Relative light units (RLU) were normalized on protein content and on background activity of controls (pGL3-Basic). Values obtained in control cells were set as 100% and at least three independent experiments each performed in triplicate were executed.

### Luciferase reporter gene assays

Cells were washed once with PBS (without Ca^2+^ and Mg^2+^) and then lysed in 100μl lysis buffer (100mM Tris/HCl (pH 7.8), 1M dithiotreitol (DTT), 0.18mM DCTA, 0.2% Triton X-100, 20% glycerol). After shaking for 30min at 30°C, samples were centrifuged (14.000rpm, 15min, 4°C) and supernatants were kept. Luciferase activities were measured according to the manufacturer’s instructions (Orion luminometer) using assay buffer (100mM KPO_4_, 15mM MgSO_4_, 4mM ATP) and D-luciferin (0.26 mg/ml; Roche Diagnostics, Indianapolis, IN, USA) dissolved in assay buffer.

### gag p19 ELISA

5x10^5^ of the respective cells were seeded and incubated for 48h at 37°C. Cells were centrifuged (1200rpm, 5min, 25°C), pellets were used for western blot analysis, and supernatants of MT-2 cells or of co-cultures from experiments using the single-cycle replication-dependent reporter vectors (see Infection assays) were sterile filtrated, and virus release was measured using gag p19 ELISA according to the manufacturer’s protocol (ZeptoMetrix Corporation, Buffalo, NY, USA). MT-2 cells were either left untreated or treated with DMSO (solvent control), cytochalasin D or nocodazole (5μM each) 48h prior to harvest. Additionally, MT-2 cells that stably carry shRNAs (shNonsense or shFascin5) were analyzed. Data were obtained using Softmax Pro Version 5.3 software (MDS Analytical Technologies, Sunnyvale, California, USA). At least, four independent experiments were performed.

### Immunoblots

Cells were washed once with PBS and protein lysates were obtained by lysis of cells in 100μl lysis buffer (150mM NaCl, 10mM Tris/HCl (pH 7.0), 10mM EDTA, 1% Triton X-100, 2mM DTT and protease inhibitors leupeptin, aprotinin (20μg/ml each) and 1mM phenylmethylsulfonyl fluoride (PMSF; 1mM)). After repeated freeze-and-thaw cycles, lysates were centrifuged (14.000rpm, 15min, 4°C). For detection of Tax, samples were sonicated three times for 20sec before centrifugation. Equal amounts of protein (50μg) were denatured for 5min at 95°C in sodium dodecyl sulfate (SDS) loading dye (10mM Tris/HCl (pH 6.8), 10% glycerin, 2% SDS, 0.1% bromophenol blue, 5% β-mercaptoethanol). After SDS-PAGE and immunoblotting on nitrocellulose transfer membranes (Whatmann, Protran, Whatmann GmbH, Dassel, Germany), proteins were detected using the following antibodies: rabbit polyclonal antibodies anti-V5 (Sigma), mouse monoclonal antibodies anti-Fascin (55K-2; Dako Deutschland GmbH, Hamburg, Germany), anti-β-actin (ACTB; Sigma), anti-Hsp90 α/β (F-8; Santa Cruz Biotechnology, Heidelberg, Germany), anti-HTLV-1 gag p19 (ZeptoMetrix Corporation), and anti-GFP (Sigma), and mouse antibodies to Tax, which were derived from the hybridoma cell line 168B17-46-34 (provided by B. Langton through the AIDS Research and Reference Reagent Program, Division of AIDS, NIAID, NIH; [45]). Secondary antibodies conjugated with horseradish peroxidase (HRP; GE Healthcare, Little Chalfont, UK) were used. Peroxidase activity was detected by enhanced chemiluminescence (ECL; 98.9% ECL A, 1% ECL B, 0.031% H_2_O_2_) using a CCD camera (Kodak Image Station 4000MM Pro camera, Kodak or Fujifilm LAS-1000 Intelligent Dark Box; Fujifilm). One of at least three independent western blots per experiment is shown. Intensities of specific bands were quantitated using Advanced Image Data Analyser (AIDA Version 4.22.034, Raytest Isotopenmessgeräte GmbH, Straubenhardt, Germany), and values were normalized on those of the housekeeping gene Hsp90 α/β.

### Quantitative real-time RT-PCR (qPCR)

10^7^ Jurkat T-cells were transfected with 50μg pEFTax or pEF. 5x10^5^ 293T cells were transfected with 2μg of pEFTax or pEF (see Transfections). 48h later, total cellular RNA was isolated from transfected Jurkat or 293T cells (RNA isolation Kit II, Macherey-Nagel, Düren, Germany) and reversely transcribed to cDNA using SuperScript II and random hexamer primers (both Life Technologies GmbH). 200ng of cDNA and SensiMix II Probe Kit (BioLine GmbH, Luckenwalde, Germany) were used according to the manufacturer’s instructions for quantitative real-time RT-PCR (qPCR) in an ABI Prism 7500 Sequence Analyzer (Applied Biosystems, Foster City, CA, USA). Primers and FAM (6-carboxyfluorescein) / TAMRA (tetramethylrhodamine)-labeled probes for detection of *β-actin* (ACTB) and *Tax* transcripts have been described before [46]. A TaqMan Gene Expression Assay (Hs00979631_g1; Applied Biosystems) was used for quantitation of Fascin transcripts. Expression levels were computed by interpolation from standard curves generated from plasmids carrying the respective target sequences and calculation of the mean of triplicated samples. Relative copy numbers (rcn) were determined by normalizing copy numbers on those of *ß-actin* (*ACTB*). At least, three independent experiments were performed.

### Immunofluorescence and confocal laser scanning microscopy

#### Subcellular localization of nanobodies, Fascin and HTLV-1 gag

5x10^5^ 293T cells were seeded on coverslips 24h prior to co-transfection with the HTLV-1 packaging plasmid pCMVHT1-ΔEnv (Δenv) (1μg) and 1μg of the respective V5-tagged nanobody expression construct (GFPNb, FASNb2, FASNb5; see Expression constructs [43]). After 48h, media were removed and cells were incubated with 250nM MitoTracker Orange CMTMRos (Life Technologies GmbH) for 30min at 37°C. Cells were washed three times in PBS, fixed with 2% paraformaldehyde (PFA; 1h, 25°C) and permeabilized with PBS/0.2% Triton X-100 (20min, 4°C). After three washing steps, unspecific binding was blocked by PBS/5% FCS/1% BSA (1h, 25°C). Samples were incubated with rabbit monoclonal antibodies anti-V5 (Sigma) and with mouse monoclonal antibodies anti-Fascin (Dako) or anti-gag p19 (ZeptoMetrix Corporation) for 30min at 37°C. After three washing steps, cells were incubated with anti-rabbit AlexaFluor488 and anti-mouse AlexaFluor647 dye-conjugated secondary antibodies (Life Technologies GmbH) for 30min at 37°C. Cells were washed three times with PBS and mounting of the samples was performed with Prolong Gold reagent with DAPI (Life Technologies GmbH) according to the manufacturer’s protocol. Images were acquired using a Leica TCS SP5 confocal laser scanning microscope equipped with a 63x1.4 HCX PL APO CS oil immersion objective lens (Leica). Images were analyzed using LAS AF software (Leica). At least 10 visual fields per condition were analyzed. Delocalization of Fascin was quantitated by comparing mean fluorescence intensities of Fascin in mitochondria with those measured in the rest of the cell.

#### Subcellular localization of Fascin and HTLV-1 gag in co-cultures of MT-2 cells or MS-9 cells with Jurkat T-cells

10^6^ Jurkat T-cells were pre-stained with 0.5μM Calcein-AM (Life Technologies GmbH) for 30min at 37°C and washed five times with PBS. Subsequently, pre-stained Jurkat T-cells were co-cultured with 10^6^ MT-2 cells that carried shFascin5 or shNonsense. In other experiments, pre-stained Jurkat T-cells were co-cultured with 10^6^ MS-9 cells. Co-cultures were seeded on poly-L-lysine- (100μg/ml; Sigma) coated coverslips and incubated for up to 1h at 37°C as indicated. Co-cultures were fixed with 2% PFA (15min, 25°C), washed three times with PBS and permeabilized with PBS/0.2% Triton X-100 (20min, 4°C). After three washing steps, unspecific binding was blocked by PBS/5% FCS/1% BSA (1h, 25°C). Samples were incubated with rabbit monoclonal antibodies anti-Fascin (Abcam, Cambridge, UK) or mouse monoclonal antibodies anti-gag p19 (ZeptoMetrix Corporation) for 30min at 37°C. After three washing steps, cells were incubated with anti-rabbit AlexaFluor555 and anti-mouse AlexaFluor350 dye-conjugated secondary antibodies (Life Technologies GmbH) for 30min at 37°C. Cells were washed three times with PBS and mounting of the samples was performed with Prolong Gold reagent without DAPI (Life Technologies GmbH) according to the manufacturer’s protocol. Images were acquired using a Leica TCS SP5 confocal laser scanning microscope equipped with a 63x1.4 HCX PL APO CS oil immersion objective lens (Leica). Images were analyzed using LAS AF software (Leica). At least 10 visual fields per condition were analyzed in three independent experiments. In co-cultures between MS-9 and Jurkat T-cells, 472 or 750 MS-9/Jurkat T-cell-conjugates seeded on fibronectin or poly-L-lysine, respectively, were analyzed. Additionally, the frequency and length of protrusions between two cells in co-cultures on poly-L-lysine-coated coverslips were measured using the LAS AF software (Leica), and the mean length of protrusions (MS-9:Jurkat: n = 50; MT-2:Jurkat: n = 83) ± standard error (SE) was calculated.

#### Automatic image analysis and measurements of cell-cell aggregation

In co-cultures of MT-2 and Jurkat T-cells (see above), we analyzed cell-cell aggregation and adhesion (see S1 Fig; [47]). For this purpose, a new image processing pipeline was developed, which enables the detection and segmentation of Jurkat T-cells in the Calcein-AM channel (life cell dye) and of MT-2 cells in the AlexaFluor350 channel (gag p19). First, candidates for Jurkat T-cells were detected and labeled using a Hough voting filter approach (S1a Fig). Using these labels as seed points, a cell segmentation was applied based on an active contours (so-called “snakes”; [48]), whose energy function has been extended by an additional energy term based on an active shape model in such a way, that the contours of adjacent and overlapping cells are resolved (S1b Fig). The active shape model energy term has been obtained from a hand-labeled reference set of cells. Due to the cross-talk between the Calcein and the AlexaFluor dyes, for segmentation of the MT-2 cells, the regions of Jurkat T-cells, which have been detected within the previous steps, were masked out to prevent to segment them as MT-2 cells. Then, a similar approach of cell detection with the Hough voting filter and segmentation based on active contours with the novel energy term was applied on the MT-2 cells (S1c–S1e Fig). In evaluation steps, the segmented cell regions in the Calcein-AM channel and the AlexaFluor350 channel were counted to obtain the number of Jurkat T-cells and MT-2 cells. Also based on these regions, the contacts between Jurkat T-cells and MT-2 cells were identified and counted. The mean number of cell-cell contacts and of adherent cells ± standard error (SE) of about 15.000 cells (~6350 MT-2; ~9650 Jurkat) of 30 different visual fields per experimental condition obtained in three independent experiments were calculated.

### Flow cytometry based assays

#### Cell-cell aggregation assay

Flow cytometry-based cell-cell aggregation assays were performed as described previously. Briefly, 10^7^ Jurkat T-cells were transfected with 30μg Tax-expression plasmids (GFP-Tax, pEFTax) and 20μg pEF, shNonsense, shFascin5 or shFascin4 (see Transfections). For cell-cell aggregation measurements, 10^6^ of transfected Jurkat T-cells were co-cultured with 10^6^ Raji/CD4^+^ B-cells for 1h at 37°C. Cells were washed with PBS and fixed with 2% PFA (15min, 25°C). Unspecific binding was blocked by washing once with PBS/5% FCS. Then, cells were incubated with anti-CD3-AlexaFluor700 (BioLegend, San Diego, CA, USA) in PBS/5% FCS to stain Jurkat T-cells and with anti-HLA-DR-PacificBlue (BioLegend) in PBS/5% FCS to stain Raji/CD4^+^ B-cells (10min, 4°C). Detection of GFP of the co-transfected shRNA constructs was used to differentiate between transfected (Tax-positive) and untransfected (Tax-negative) cells. Afterwards, the percentage of double-stained cells (CD3^+^/HLA-DR^+^), representing cell-aggregates of Jurkat T-cells with Raji/CD4^+^ B-cells, was determined with the BD LSRII flow cytometer (BD Biosciences, San Jose, CA, USA) and normalized on the percentage of CD3^+^ cells (Jurkat T-cells).

#### Measurement of gag transfer

10^6^ MT-2 cells were co-cultured with 10^6^ Jurkat T-cells for 1h at 37°C. After washing with PBS, cells were fixed with 2% PFA (15min, 25°C) and permeabilized using saponin: cells were incubated with mouse monoclonal antibodies anti-gag p19 (ZeptoMetrix Corporation) in PBS/0.3% saponin (30min, 4°C) and washed with PBS/0.1% saponin. Cells were incubated with anti-mouse AlexaFluor647-conjugated secondary antibodies (LifeTechnologies GmbH) in PBS/0.3% saponin (30min, 4°C) and washed twice with PBS/0.1% saponin. After washing with PBS/5% FCS, cells were incubated with anti-CD25-PE (Miltenyi Biotech GmbH, Bergisch-Gladbach, Germany) in PBS/5% FCS to stain MT-2 cells (10min, 4°C). Cells were analyzed using the BD LSR II flow cytometer (BD Biosciences). Cells were discriminated by their different size (FSC/SSC) and by CD25-staining. The percentage of gag-positive cells within CD25-negative cells (Jurkat T-cells) was examined to measure gag transfer and infection rates from MT-2 to Jurkat T-cells.

#### Measurement of apoptosis and cell death

10^7^ Jurkat T-cells were transfected with 30μg pEFTax or pEF and 20μg shNonsense, shFascin5 or shFascin4 (see Transfections). Cells treated with the topoisomerase II inhibitor etoposide (15μM) served as positive control for cell death, DMSO as solvent control. Jurkat T-cells were stained 48h after transfection with AnnexinV/Sytox using Violet AnnexinV/Dead Cell Apoptosis Kit with Pacific Blue AnnexinV/Sytox AADvanced for Flow Cytometry (Life Technologies GmbH) according to the manufacturer’s instructions. The percentage of apoptotic (AnnexinV^+^; Sytox^-^), late apoptotic (AnnexinV^-^; Sytox^+^) or dead (AnnexinV^+^; Sytox^+^) cells was determined using BD LSRII flow cytometer (BD Biosciences). As fixation with 2% PFA is not compatible with the dye Sytox, HTLV-1-infected MT-2 and HuT-102 cells and stable 293T cells (shNonsense; shFascin4; shFascin5) were stained with AnnexinV (from the above mentioned kit) and with LiveDead (LIVE/DEAD Fixable Far Red Stain Dead Cell Stain Kit, Life Technologies GmbH) instead of Sytox according to the manufacturer’s instructions. Apoptosis and cell death were determined as described above.

### Statistics

Microsoft Office Excel software was used for statistical analysis using the t-test (unpaired). P<0.05 was considered to be significant.

## Results

### Endogenous Fascin is important for transmission of HTLV-1 reporter vectors

**Fig 1:**
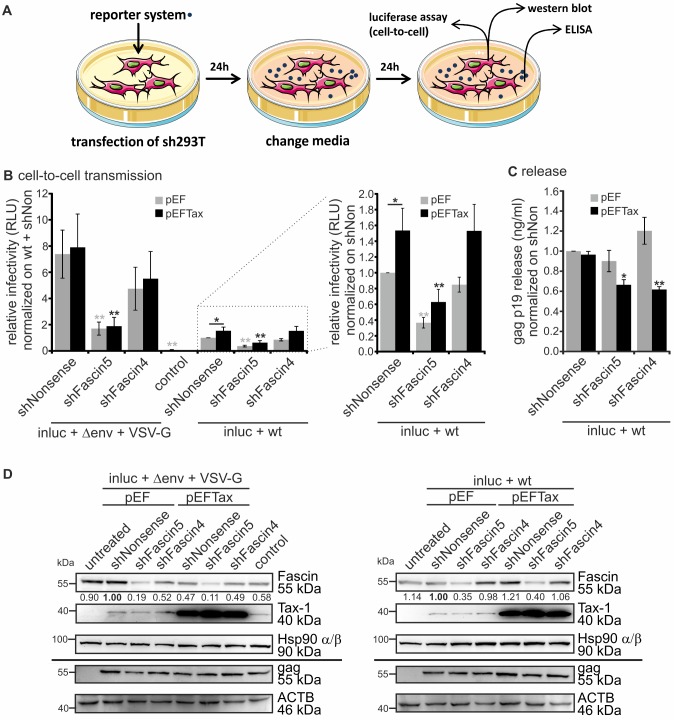
Repression of endogenous Fascin impairs virus release and HTLV-1 reporter activity independent of the envelope type used. **(A)** Scheme of experimental setup using single-cycle replication-dependent reporter vectors with 293T cells. **(A-D)** Stable 293T cells (sh293T) that carry one of two different shRNAs targeting Fascin (shFascin5, shFascin4) or the control (shNonsense) were transfected with the reporter vector pCRU5HT1M-inluc (inluc) and the packaging plasmids pCMVHT1M containing either HTLV-1 wildtype env (wt) or lacking env (Δenv). The latter were pseudotyped with VSV-G or supplemented with pcDNA3 (control). Cells were co-transfected with pEFTax or pEF (mock). Luciferase assays, ELISA and western blot were performed as shown in **A)**. Values were normalized on those obtained from shNonsense 293T cells transfected with inluc+wt, and the mean of four independent experiments ± standard error (SE) is shown. Values were compared to the respective mock (shNonsense+pEF or shNonsense+pEFTax) using Student’s t-test (*: p<0.05, **: p<0.01). **(B)** Luciferase activity (cell-to-cell transmission). Right panel: enlargement of dotted box. **(C)** Detection of gag p19 release by ELISA. **(D)** Detection of Fascin, Tax-1 and gag by western blot. Hsp90 α/β and β-actin (ACTB) served as control. Numbers indicate densitometric analysis of Fascin detection normalized on Hsp90 α/β.

To assess the role of Fascin on HTLV-1 transmission, we made use of a single-cycle replication-dependent reporter system that is transfected into donor cells and allows monitoring of reporter gene activity in newly infected target cells only [25]. Briefly, a virus packaging plasmid encoding all HTLV-1 genes (wildtype; wt) and a replication-dependent HTLV-1 reporter vector containing a CMV-promoter driven *luciferase* (*luc*) gene were co-transfected into 293T cells. Alternatively, an HTLV-1 packaging plasmid carrying a mutation in the *envelope* (*env*) gene, and a VSV-G-expression plasmid were co-transfected instead of wt. The *luc* gene is oriented in antisense and is interrupted by an intron oriented in sense, therefore translation of the reporter mRNA in transfected cells is precluded. The vector mRNA is spliced and packaged into VLPs. After infection and replication, a provirus that lacks the intron is generated, and reporter gene expression (luc activity) can be measured in the target cell [25]. We previously used this system to assess the role of cellular restriction factors on HTLV-1 [49]. To analyze whether Fascin is important for transmission of these HTLV-1 reporter vectors, stable 293T cells with a knockdown of Fascin were generated. For this purpose, cells were transfected with two different shRNA constructs carrying an IRES-EGFP expression cassette and shRNAs targeting Fascin (shFascin5, shFascin4; [29,42]) or a control (shNonsense), and cells were selected with puromycin. Flow cytometry monitoring GFP-expression revealed that approximately 90% of cells carried the shRNA-constructs (S2A Fig). Beyond, vitality of stable cell lines was unaffected by the presence of shRNAs as detected by live/dead staining (S2B Fig). Cell lines were transfected with single-cycle replication-dependent HTLV-1 reporter vectors (inluc), a viral packaging plasmid (Δenv or wt), and as indicated with VSV-G for pseudotyping (Fig 1A). sh293T cells (shNonsense) transfected with inluc and Δenv served as negative control (control, Fig 1B) for both wt env-carrying and VSV-G-pseudotyped viral particles. After 24h, media were changed, and after another 24h, cells were harvested to measure cell-to-cell transmission in luciferase assays (Fig 1B), virus release by gag p19 ELISA (Fig 1C) and protein expression by western blot analysis (Fig 1D).

Making use of single-cycle replication-dependent HTLV-1 reporter vectors revealed that stable repression of endogenous Fascin by shRNAs leads to a significant reduction of reporter gene activity (Fig 1B). While shFascin5 resulted in a strong reduction of reporter gene activity by more than 70% (Fig 1B) and, in parallel, of Fascin protein (Fig 1D), the influence of shFascin4 on reporter gene activity (Fig 1B; by 30%) and on Fascin protein expression (Fig 1D) was less pronounced. Overexpression of Tax (black bars) did not enhance transmission of VSV-G-pseudotyped HTLV-1 (Fig 1B, left part of left panel), confirming earlier observations [25]. However, overexpressed Tax enhanced cell-to-cell-transmission of HTLV-1 reporters packaged with wt env (Fig 1B, right part of left panel and enlargement in right panel) contrary to previous observations [25]. The latter suggests that Tax and wt env cooperate in cell-to-cell transmission. The relative infectivity of VSV-G pseudotyped reporter vectors was about 7-fold higher than that of wt env-pseudotyped reporter vectors (Fig 1B) and undetectable, if no envelope was added (control). However, independent of the envelope type used, repression of Fascin significantly reduced the relative infectivity of both wt env-carrying and VSV-G-pseudotyped reporter vectors. Moreover, independent of the experimental condition, Tax could not further induce expression of Fascin protein in 293T cells (Fig 1D). While Tax led to a robust induction of Fascin mRNA (S4A Fig) and protein (S4B Fig) in Jurkat T-cells confirming our previous work [29], Tax did not further modulate Fascin expression in 293T cells, which already exhibit high amounts of endogenous Fascin (S4A and S4B Fig). Yet, reporter gene activity as a measure of HTLV-1 cell-to-cell transmission was Fascin-dependent in presence of overexpressed Tax, too (Fig 1B), suggesting that Fascin is also important for HTLV-1 cell-to-cell transmission in cells which express high amounts of endogenous Fascin. To analyze whether Fascin also impairs virus release, the viral gag p19 protein was measured by ELISA in cells that had been transfected with HTLV-1 reporters packaged with wt env. Overexpression of Tax (Fig 1C, black bars) did not further enhance gag p19 levels in the supernatants of 293T cells and repression of Fascin led to approximately 40% reduction of virus release only when Tax was supplemented suggesting that Tax and Fascin cooperate in processes that are important for virus release. Since repression of Fascin led to a severe defect of cell-to-cell transmission also in absence of supplemented Tax (Fig 1B, grey bars), but not to a decrease in virus release (Fig 1C, grey bars), this suggests that Fascin’s role in cell-to-cell transmission dominates over its role on virus release in this experimental setup. However, results obtained by the reporter system only provide a signal upon productive infection of a target cell, while the gag p19 ELISA also quantifies non-infectious VLPs. To exclude that virus production in the cell is already impaired by repression of Fascin, western blots detecting gag were performed (Fig 1D). Overall levels of cell-associated gag p55 were comparable between different experimental conditions. Beyond, Fascin was strongly repressed in presence of shFascin5 and only moderately repressed in presence of shFascin4. We could also detect Tax expressed from the packaging plasmids as tiny band, and an increased expression of Tax upon supplementing a Tax expression plasmid. Taken together, our data indicate that Fascin is important for transmission of HTLV-1 reporter vectors independent of the envelope type in 293T cells.

### Fascin nanobodies block transmission of HTLV-1 reporter vectors

**Fig 2:**
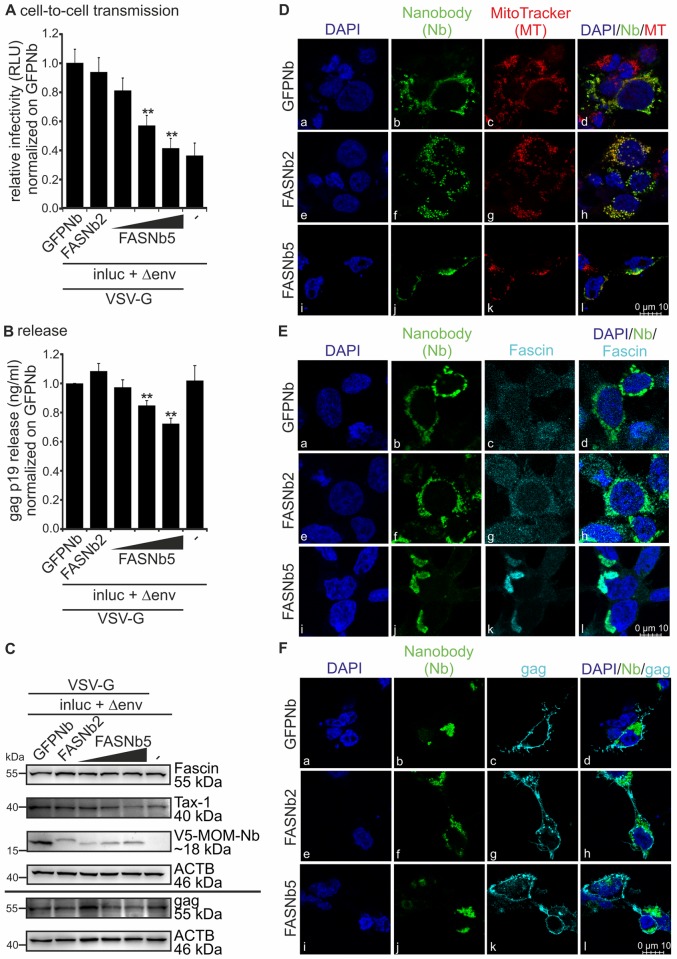
Inhibition of actin-bundling using Fascin-specific nanobodies displays a significant reduction of virus transmission. **(A-C)** 293T cells were transfected with the reporter vector pCRU5HT1M-inluc (inluc) and the packaging plasmid pCMVHT1M-ΔEnv encoding all HTLV-1 proteins except env (Δenv) pseudotyped with VSV-G. Cells were co-transfected with V5-tagged expression plasmids encoding a mitochondrial outer membrane (MOM) sequence and nanobodies (Nb) targeting Fascin (MOMFASNb2 (FASNb2), 0.5μg; or MOMFASNb5 (FASNb5), 0.25; 0.5; 1μg), or a control nanobody (MOMGFPNb (GFPNb), 0.5μg). At 48h post transfection, luciferase assays, ELISA and western blot were performed as described in Fig 1A. The means of four independent experiments ± standard error (SE) are shown and were compared to the control (GFPNb) using Student’s t-test (**: p<0.01). **(A)** Luciferase activity (cell-to-cell transmission). **(B)** Detection of gag p19 release by ELISA. **(C)** Detection of Fascin, Tax-1, V5 (MOM-Nanobodies) and gag by western blot. β-actin (ACTB) served as control. **(D-F)** Confocal laser scanning microscopy of 293T cells co-transfected with pCMVHT1M-ΔEnv and with V5-tagged expression plasmids encoding a MOM-sequence and nanobodies FASNb2, FASNb5 or GFPNb (control). Stains of nanobodies (V5; green) and **(D)** mitochondria (red), **(E)** Fascin (turquoise), and **(F)** gag p19 (turquoise) and the merges of the respective stains are shown. DAPI (blue) served as control.

To strengthen our results, we made use of Fascin-specific nanobodies that had been developed and characterized previously [43]. Briefly, nanobodies are antigen-binding domains of camelid heavy-chain antibodies. The employed Fascin-specific nanobodies contain a mitochondrial outer membrane (MOM) signal that leads to targeted subcellular delocalization of Fascin to the MOM [43]. Use of these nanobodies allowed us to trigger Fascin protein loss of function without changing its expression. Upon transfection of HTLV-1 reporter vectors and expression plasmids encoding Fascin-specific nanobodies into 293T cells, luciferase assays (Fig 2A), gag p19 ELISA (Fig 2B), western blot analysis (Fig 2C) and immunofluorescence stains followed by confocal laser scanning microscopy (Fig 2D–2F) were performed. We found that Fascin nanobody 5 (FASNb5) significantly reduced reporter gene activity in a dose-dependent manner compared to a control nanobody (GFPNb) and to FASNb2 (Fig 2A). In gag p19 ELISA (Fig 2B), we measured a dose-dependent decrease of released gag p19 into the supernatants in presence of FASNb5, suggesting that this nanobody also impairs release of HTLV-1. Expression of the nanobodies and the unaltered expression of Fascin were confirmed by western blot analysis (Fig 2C). Additionally, immunofluorescence was performed confirming earlier studies [43] showing co-localizations of V5-tagged and MOM-expressing nanobodies (GFPNb, FASNb2, FASNb5) with mitochondria (Fig 2D). Next, we checked whether Fascin-specific nanobodies lead to efficient delocalization of Fascin by staining V5-tagged nanobodies and Fascin. Immunofluorescence analysis revealed that FASNb5 lead to a more efficient delocalization of Fascin (90.2% of Fascin delocalized) compared to FASNb2 (71.1%; Fig 2E), which mirrors the different impact of FASNb5 and FASNb2 on cell-to-cell transmission (Fig 2B) and virus release (Fig 2C). However, since FASNb5 also impairs Fascin-mediated actin-bundling compared to FASNb2 [43], these data also suggest that Fascin’s actin-bundling activity could be required for transmission of HTLV-1. Contrary to delocalizing Fascin, FASNb5 did not delocalize gag to the mitochondria (Fig 2F), suggesting that Fascin and gag do not directly interact, or, if they interact, the interaction is not sustained during delocalization. Moreover, the impact of FASNb5 on virus release as measured by gag p19 ELISA may be indirect (Fig 2B), e.g. by impairing the transport of gag to budding sites or by impairing budding itself. Summed up, not only repression, but also delocalization of Fascin in the cell interferes with HTLV-1 cell-to-cell transmission.

### Tax-induced Fascin is important for release and cell-to-cell transmission of HTLV-1 reporter vectors

**Fig 3:**
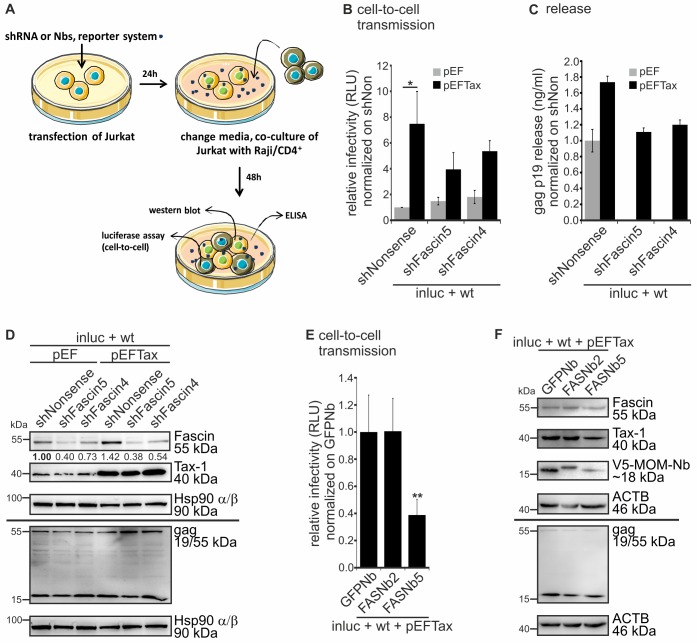
Repression of Tax-induced Fascin results in reduction of both virus release and cell-to-cell transmission. **(A)** Scheme of experimental setup using single-cycle replication-dependent reporter vectors in Jurkat T-cells and Raji/CD4^+^ B-cells. **(A-D)** Jurkat T-cells were transfected with the reporter vector pCRU5HT1M-inluc (inluc) and the packaging plasmid pCMVHT1M encoding HTLV-1 with wildtype env (wt). Cells were co-transfected with pEFTax or pEF (mock) and one of two different shRNAs targeting Fascin (shFascin5, shFascin4) or the control (shNonsense). Luciferase assays, ELISA and western blot were performed as depicted in **A)**. **(B)** Luciferase activity (cell-to-cell transmission). The means of four independent experiments ± standard error (SE) are shown and were compared to the respective mock (shNonsense+pEF or shNonsense+pEFTax) using Student’s t-tests (*: p<0.05). **(C)** Detection of gag p19 release by ELISA. A representative experiment is shown. **(D)** Detection of Fascin, Tax-1 and gag by western blot. Hsp90 α/β served as control. Numbers indicate densitometric analysis of Fascin detection normalized on Hsp90 α/β. **(E-F)** Jurkat T-cells were co-transfected with the reporter vector pCRU5HT1M-inluc (inluc), the packaging plasmid pCMVHT1M (wt), pEFTax, and one of three different V5-tagged expression plasmids encoding a MOM-sequence and nanobodies FASNb2, FASNb5 or GFPNb (control). Luciferase assays and western blot were performed as depicted in **A)**. **(E)** Luciferase activity (cell-to-cell transmission). The means of six independent experiments ± standard error (SE) were normalized and compared to control samples (GFPNb) using Student’s t-tests (**: p<0.01). **(F)** Detection of Fascin, Tax-1, V5-tagged nanobodies and gag by western blot. β-actin (ACTB) served as control.

Our results obtained thus far do not exclude that repression of Fascin impairs viral entry since we used a one-step transfection/infection co-culture system, where transfected cells produce VLPs that infect neighboring cells [25], which also harbor a knockdown of Fascin, or which could be impaired by Fascin-specific nanobodies. Further, since HTLV-1 predominantly infects CD4^+^ T-cells *in vivo*, we switched to a more physiological system and analyzed the role of Tax and Fascin on HTLV-1 transmission in CD4^+^ Jurkat T-cells in co-culture with Raji/CD4^+^ B-cells, a co-culture system that had been described earlier to allow monitoring of HTLV-1 transmission with single-cycle replication-dependent reporter vectors [25]. Upon co-transfection of Jurkat T-cells with HTLV-1 reporters (inluc), packaging plasmids (wt), Tax expression plasmids and shRNAs targeting Fascin (Fig 3A), media were changed at 24h, and Jurkat T-cells were co-cultured for another 48h with Raji/CD4^+^ B-cells. Measurement of luciferase reporter gene activity reflecting cell-to-cell transmission revealed that repression of Fascin did not affect basal HTLV-1 cell-to-cell transmission (Fig 3B, grey bars). However, upon overexpression of Tax (black bars), reporter gene activity significantly increased confirming earlier observations in this cell type [25]. Interestingly, repression of Fascin led to a reduction of Tax-induced reporter gene activity suggesting that Fascin is a major contributor of Tax-induced cell-to-cell transmission. To exclude and to confirm that the measured reporter gene activity is not due to cell-free virus transmission, we incubated Raji/CD4^+^ B-cells with supernatants of Jurkat T-cells that had been transfected with the reporter system, which did not result in detectable luciferase signals (S3 Fig; [25]). Measuring of gag p19 release by ELISA mirrored the results obtained by luciferase assays and showed that Tax-enhanced virus release in Jurkat T-cells occurs Fascin-dependently (Fig 3C). Knockdown of Fascin in presence of overexpressed Tax led to a reduction of gag p19 release nearly reaching those levels measured without supplemented Tax. Western blot analysis showed that, contrary to 293T cells (Fig 1D, S4 Fig), Tax is a potent inducer of Fascin transcript and protein expression in Jurkat T-cells (Fig 3D, S4 Fig) confirming our previous results [29,34]. Thus, too low levels of endogenous Fascin in Jurkat T-cells without overexpressed Tax (S4 Fig) could be a potential explanation for the Tax-dependency of the Fascin-effect in this cell type. Concomitant with our findings obtained in 293T cells (Fig 1D), western blot analysis revealed that the levels of cell-associated gag p55 were comparable between different experimental conditions also in Jurkat T-cells (Fig 3D). Contrary to 293T cells, Fascin was induced by Tax in Jurkat T-cells. Further, Fascin was strongly repressed in presence of shFascin5 and moderately repressed in presence of shFascin4. Tax expressed from the packaging plasmids could be detected as a tiny band, and an increased expression of Tax was detectable upon supplementing a Tax expression plasmid. Thus, Tax enhances virus release, and augments cell-to-cell transmission from Jurkat T-cells to Raji/CD4^+^ B-cells dependent on Fascin. To substantiate these findings, we tested Fascin-specific nanobodies instead of shRNAs in the Jurkat-Raji/CD4^+^ co-culture model. Compared to the control nanobody GFPNb and to FASNb2, FASNb5 led to a significant reduction (by 61%) of Tax-induced HTLV-1 reporter gene activity (Fig 3E). This suggests that delocalization of Fascin without changing its expression (Fig 3F) and inhibition of Fascin’s actin-bundling activity by FASNb5 [43] impair HTLV-1 cell-to-cell transmission in different cell types (Figs 2, 3E and 3F). Taken together, use of single-cycle replication-dependent HTLV-1 reporter vectors revealed that stable repression of endogenous Fascin (293T cells), or of Tax-induced Fascin (Jurkat T-cells) by shRNAs and inhibition of Fascin using specific nanobodies impair both gag p19 release and HTLV-1 cell-to-cell transmission.

### Repression of Fascin in HTLV-1-infected T-cells reduces transactivation and infection of co-cultured T-cells

**Fig 4:**
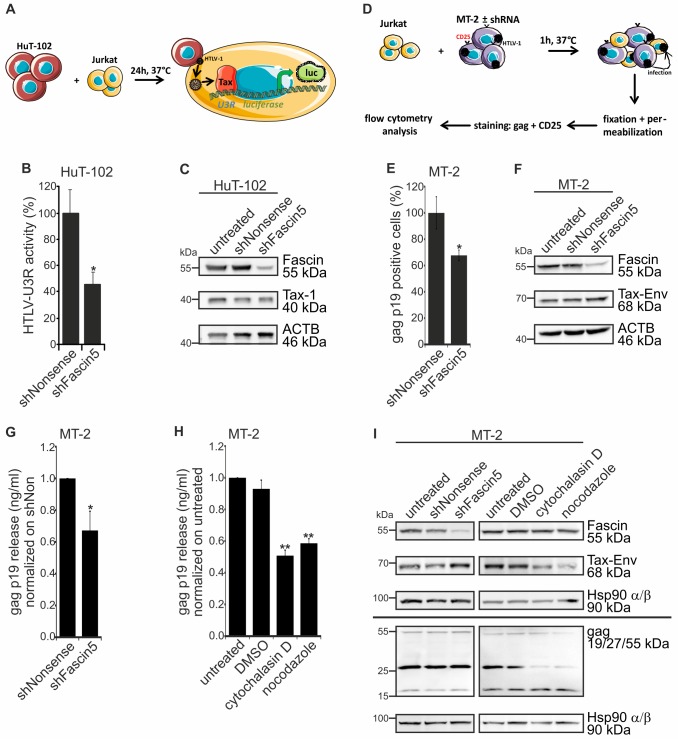
Fascin knockdown in chronically HTLV-1-infected T-cells impairs virus release and infection of co-cultured T-cells. **(A)** Scheme of co-culture experiments using HuT-102 cells and reporter Jurkat T-cells. **(A-C)** Chronically HTLV-1-infected HuT-102 cells with stable repression of Fascin (shFascin5) or control cells (untreated, shNonsense) were co-cultured with Jurkat T-cells that had been transfected 24h earlier with luciferase reporter vectors carrying the core promoter U3R of HTLV-1 (pGL3-U3R) or a control (pGL3-Basic). After 48h, relative light units (RLU) normalized on protein content and on background activity (pGL3-Basic) were determined. **(B)** Luciferase activity of co-cultures. The means of four independent experiments ± standard error (SE) are shown and compared to shNonsense using Student’s t-test (*: p<0.05). **(C)** Detection of Fascin and Tax-1 by western blot. β-actin (ACTB) served as control. **(D)** Scheme of infection experiments using MT-2 cells and Jurkat T-cells. **(D-F)** Chronically HTLV-1-infected MT-2 cells with stable repression of Fascin (shFascin5) or control cells (untreated, shNonsense) were co-cultured with Jurkat T-cells for 1h at 37°C. Thereafter, cells were stained for CD25 and gag p19 and analyzed by flow cytometry to detect the number of newly-infected Jurkat T-cells (CD25^-^ gagp19^+^). **(E)** Gag-positive Jurkat T-cells (%) co-cultured with the respective MT-2 cells. The means of four independent experiments ± standard error (SE) are shown and compared to shNonsense using Student’s t-test (*: p<0.05). **(F)** Detection of Fascin and Tax-Env by western blot. β-actin (ACTB) served as control. **(G-H)** Gag p19 ELISA using supernatants of **(G)** stable MT-2 cells (shNonsense, shFascin5) and **(H)** differently treated MT-2 cells. Equal numbers of cells (10^5^ cells/ml) were seeded and treated with cytochalasin D, nocodazole (each 5μM), or DMSO (control) for 48h. The means of at least four independent experiments ± standard error (SE) are shown and were compared to control cells (shNonsense or untreated) using Student’s t-test (*: p<0.05, **: p<0.01). **(I)** Detection of Fascin, Tax-Env and gag by western blot. Hsp90 α/β served as control.

Next, we asked whether Fascin contributes to HTLV-1 cell-to-cell transmission also in chronically HTLV-1-infected T-cells, which express high amounts of Fascin protein. For this purpose, ATL-derived HTLV-1-infected HuT-102 cells were stably transduced with lentiviral vectors expressing either a shRNA targeting Fascin (shFascin5) or a nonsense shRNA (shNonsense) [29]. According to a published protocol [50], HuT-102 cells were co-cultured with Jurkat T-cells that had been transfected with an HTLV-1-LTR (U3R)-dependent *luc* gene reporter system (pGL3-U3R; Fig 4A). Upon infection of Jurkat T-cells, the viral Tax protein should activate expression of the HTLV-1 U3R resulting in enhanced luciferase activity. After 24h of co-culture, luciferase activity was measured and normalized on protein content and on transactivation of a mock luciferase construct (Fig 4B). Transactivation of the reporter in Jurkat T-cells was diminished by more than 50% when Fascin was knocked down in the co-cultured HTLV-1-infected HuT-102 cells hinting at a role of Fascin for HTLV-1-mediated cell-to-cell transfer. In parallel, knockdown of Fascin in HuT-102 was verified in immunoblots and shown to be Fascin-specific since Tax protein and the housekeeping gene ß-actin (ACTB) were not affected by shFascin5 (Fig 4C). Further, we excluded detrimental effects of the shRNA on cell vitality by measuring apoptosis and cell death in stable cell lines compared to cells treated with 15μM etoposide, which is known to induce cell death (S5 Fig). Since results from co-culture assays may also reflect cell fusion events or the transfer of Tax-containing exosomes [51], we decided to measure HTLV-1 infection also directly by a flow cytometry based assay that allows monitoring of newly infected cells. For this purpose, we first stably transduced the chronically HTLV-1-infected T-cell line MT-2 cells with lentiviral vectors expressing either shFascin5 or shNonsense. According to an established protocol [28], MT-2 cells were then co-cultured with uninfected Jurkat T-cells for 1h and the number of newly infected Jurkat T-cells was detected by flow cytometry by measuring the amount of the viral matrix protein gag p19 in these cells (Fig 4D and 4E). For this purpose, co-cultures were permeabilized and stained with antibodies targeting HTLV-1 gag p19 and the IL-2 receptor alpha chain CD25, which is present on MT-2 cells, but not on Jurkat T-cells [52]. Flow cytometry revealed that repression of Fascin led to a significant reduction of newly infected, gag p19-positive Jurkat T-cells to 68% compared to the control (Fig 4E). Beyond, western blot analysis confirmed a robust reduction of Fascin protein in MT-2 cells carrying shFascin5 (Fig 4F), while Tax and ACTB were unaffected. Further, cell vitality was also unaffected by repression of Fascin as indicated by live/dead stainings (S5 Fig). We also measured release of gag p19 into culture supernatants and found that knockdown of Fascin not only reduced infection of co-cultured Jurkat T-cells, but also diminished the release of gag p19 (Fig 4G). Similar results were obtained with MT-2 cells treated with cytochalasin D or nocodazole (5μM each), which interfere with actin or tubulin polymerization, respectively (Fig 4H). Overall, our observations are in line with the data we obtained with the HTLV-1 reporter vectors (Figs 1–3) suggesting that, independent of the cell and test system used, repression of Fascin impairs release and cell-to-cell transmission of HTLV-1. Immunoblot analysis revealed that cell-associated gag and processing of the gag p55 precursor into gag p19 and gag p27 was unaffected by knockdown of Fascin (Fig 4I). However, treatment of MT-2 cells with the compounds cytochalasin D and nocodazole interfered with processing of gag p55 suggesting that chemical interference with the cytoskeleton acts differently on virus production than Fascin repression. Taken together, we found that Fascin is critical for release and cell-to-cell transmission of HTLV-1 reporter vectors, and for transactivation and infection of co-cultured T-cells indicating an important role of Fascin in HTLV-1 cell-to-cell transmission.

### Tax-enhanced cell-cell aggregation is independent of Fascin

**Fig 5:**
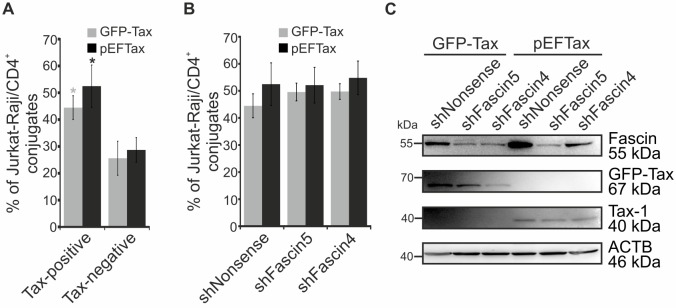
Knockdown of Fascin does not influence cell aggregation between Jurkat T-cells and Raji/CD4^+^ B-cells. **(A-C)** Conjugate formation between transfected Jurkat T-cells and co-cultured Raji/CD4^+^ B-cells was quantitated by flow cytometry. Jurkat T-cells were transfected with one of two different Tax-expression constructs (GFP-Tax; pEFTax) and one of two different shRNAs encoding IRES-EGFP and targeting Fascin (shFascin5, shFascin4) or a control (shNonsense). After 24h, Jurkat T-cells were co-cultured with Raji/CD4^+^ B-cells (ratio 1:1) for 1h at 37°C. Co-cultures were fixed and stained with anti-CD3-AlexaFluor700 (for Jurkat T-cells) and anti-HLA-DR-PacificBlue antibodies (for Raji/CD4^+^ B-cells) to differentiate between the two cell types. **(A)** Detection of GFP encoded by GFP-Tax and/or the shRNA construct (shNonsense) was used to differ between transfected (GFP^+^) and untransfected (GFP^-^) cells. Cell-cell conjugates were identified in GFP^+^ (Tax-positive) and GFP^-^ (Tax-negative) gates as double-positive signals (HLA-DR^+^CD3^+^) and normalized on the total number of Jurkat T-cells (CD3^+^; see S6 Fig). The means of four independent experiments ± standard error (SE) are shown. Student’s t-tests were performed (*: p<0.05). **(B)** Control cells (shNonsense) compared to Fascin-repressed cells (shFascin5, shFascin4) within Tax-positive cells of **A)**. **(C)** Detection of Fascin, GFP-Tax and Tax-1 in transfected Jurkat T-cells by western blot. β-actin (ACTB) served as control.

To shed light on the mechanism of Fascin’s role during HTLV-1 transmission, we asked whether Fascin enhances conjugate formation between infected and uninfected T-cells similar to the small GTP-binding protein GEM [28]. For this purpose, we performed a flow cytometry-based conjugate formation assay between Jurkat T-cells that had been transfected with Tax and Fascin-specific shRNAs as donor cells, and Raji/CD4^+^ B-cells as acceptor cells according to a previously described protocol [25]. Briefly, Jurkat T-cells were co-transfected with one of two different Tax-expression constructs (GFP-Tax; pEFTax) and one of two different shRNAs encoding IRES-EGFP and targeting Fascin (shFascin5, shFascin4) or a control (shNonsense). After 24h, cells were co-cultured with Raji/CD4^+^ B-cells for 1h and the percentage of conjugate formation between the two cell types was quantitated by flow cytometry (S6 Fig). Detection of GFP encoded by GFP-Tax and/or the shRNA constructs was used to differ between transfected (GFP^+^) and untransfected (GFP^-^) Jurkat T-cells. Conjugates of Jurkat T-cells (CD3^+^) with Raji/CD4^+^ B-cells (HLA-DR^+^) were identified in GFP^+^ (Tax-positive) and GFP^-^ (Tax-negative) gates as double-positive signals (HLA-DR^+^CD3^+^) and normalized on the total number of Jurkat T-cells. While Tax enhanced conjugate formation between the two different cell types confirming earlier observations (Fig 5A; [25]), we found that Tax-induced cell aggregation was independent of Fascin (Fig 5B). Western Blot analysis confirmed the expression of GFP-Tax, Tax, and the functionality of the Fascin-specific shRNAs (Fig 5C).

### The number of cell-cell aggregates between HTLV-1-infected and uninfected T-cells is independent of Fascin, but adhesion of infected T-cells is dependent on Fascin

**Fig 6:**
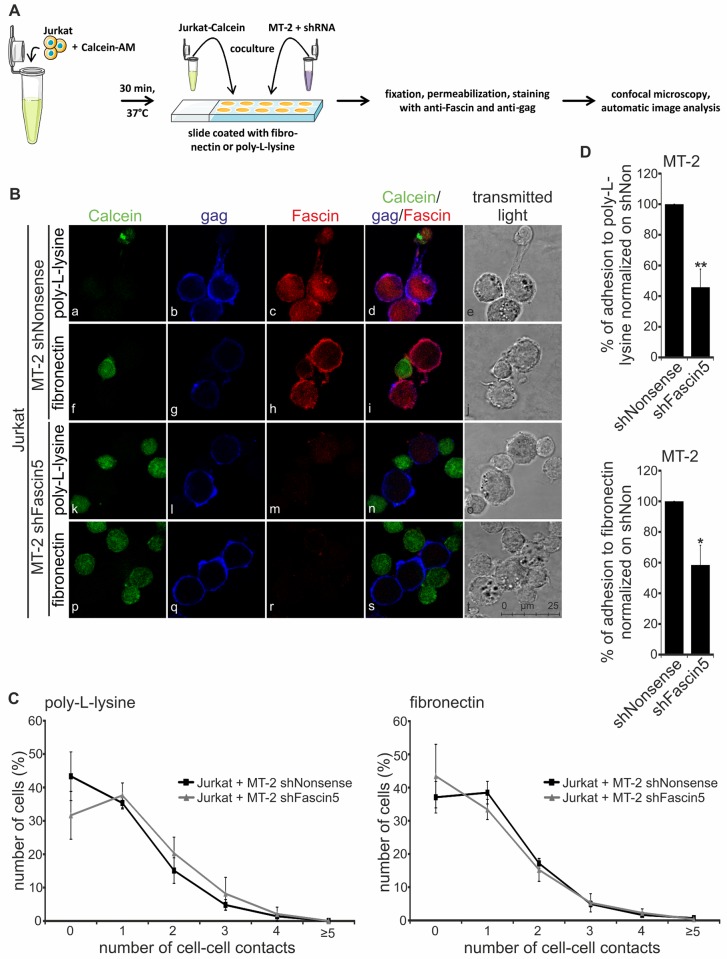
Knockdown of Fascin does not affect cell aggregation but decreases cell adhesion of MT-2 cells in co-cultures with Jurkat T-cells. **(A)** Scheme of immunofluorescence stains with MT-2 cells and Jurkat T-cells. **(A-D)** MT-2 cells with stable repression of Fascin (shFascin5) or control cells (shNonsense) were co-cultured with Calcein-stained Jurkat T-cells for 1h at 37°C either on poly-L-lysine- (a-e, k-o) or on fibronectin-coated (f-j, p-t) coverslips. **(B)** Immunofluorescence stainings of co-cultures. Jurkat cells were pre-stained with Calcein-AM (green) to differentiate between the two cell types. Stainings of gag (blue), Fascin (red) and the merge of all three stainings are shown. Transmitted light served as control. Representative stainings of three independent experiments are shown. **(C-D)** Results of automatic image analysis (see S1 Fig). The means of three independent experiments ± standard error (SE) are shown and were compared to shNonsense using Student’s t-tests (*: p<0.05, **: p<0.01). **(C)** Percentage of MT-2 cells (shNonsense, shFascin5) with 0 to ≥5 cell-to-cell contacts to co-cultured Jurkat T-cells on the different coatings. **(D)** Percentage of adherent MT-2 cells (shNonsense, shFascin5) on the different coatings normalized on MT-2 shNonsense.

To validate these findings also in chronically infected T-cells, we asked whether Fascin is important for conjugate formation between HTLV-1-infected MT-2 cells and Jurkat T-cells, or whether Fascin contributes to adhesion of HTLV-1-infected cells on different attachment factors. For this purpose, HTLV-1-infected MT-2 cells with repressed Fascin (shFascin5) and the respective controls (shNonsense, untreated) were co-cultured with Jurkat T-cells that had been pre-stained with the life cell dye Calcein-AM (green). After 1h of co-culture at 37°C, cells were spotted on glass slides either coated with poly-L-lysine or fibronectin (Fig 6A). Co-cultures were stained with antibodies targeting the viral matrix protein gag p19 (blue) to label HTLV-1-infected MT-2 cells and with antibodies targeting Fascin (red; Fig 6B).

Immunofluorescence revealed that gag was detectable in all experimental conditions, while expression of Fascin was repressed in MT-2 shFascin5 cells. Overlay of the respective channels and transmitted light showed that protrusive structures between chronically infected MT-2 cells and uninfected Jurkat T-cells could be detected (Fig 6Bd and 6Be). The length of the protrusions was approximately 8.35μm+/-2.05μm (on fibronectin) or 6.16μm+/-2.24μm (on poly-L-lysine). In most cells, Fascin and gag localized in close proximity (Fig 6Bi), and occasionally, both proteins co-localized (Fig 6Bd). This was further confirmed by determining fluorescence intensities of both Fascin and gag along arbitrary drawn ROIs (regions of interest; S7a–S7g Fig): (1) Fascin and gag localize in close proximity, but do not co-localize (ROI 1). (2) The parallel shape of Fascin and gag fluorescence intensities at ROI 2 suggests that both proteins co-localize. Since co-localization events were only found in < 5% of all MT-2 cells, our findings suggest rather a transient or indirect than a tight and direct interaction between Fascin and gag during the dynamic process of virus budding.

Next, automatic image analysis was performed to count the number of cell-cell contacts between infected MT-2 cells and uninfected Jurkat T-cells and to handle the large numbers of images and cells. An image processing algorithm was developed and applied that allowed for automatic quantitation of the respective cell types, and for counting of cell-cell contacts between infected and uninfected cells [47]. Automatic image analysis discriminated between Jurkat T-cells and MT-2 cells based on a cell segmentation approach using an active contour algorithm incorporating *a priori* shape information. The accuracy of cell segmentation determined on a single cell basis added up to *a*_J_ = 82.5% for Jurkat T-cells and *a*_M_ = 77.8% for MT-2 cells. Correctly identified Jurkat cells were segmented with a hit quality *h*_J_ = 96.4% and correctly identified MT-2 cells with *h*_M_ = 83.2%. In parallel, the micrograph images were checked manually to include cells into the evaluation that had been missed by the algorithm. Applying this algorithm, the following findings were obtained: (1) The number of cell-cell aggregates between HTLV-1-infected and uninfected T-cells is independent of the attachment factor and of Fascin (Fig 6C), confirming our results from the flow cytometry-based assay (Fig 5). (2) Adhesion of HTLV-1-infected MT-2 cells is significantly impaired upon knockdown of Fascin (Fig 6D), while cell vitality is unaffected (S5 Fig). Thus, Fascin seems to be important for proper attachment of MT-2 cells on fibronectin- and poly-L-lysine-coated matrices and could favor dissemination of infected cells *in vivo*. Taken together, although Fascin seems to be required for proper attachment, Fascin does not affect the quantity of cell-cell contacts to uninfected Jurkat cells.

### Fascin localizes at cell-cell contacts and in long-distance connections in proximity to gag

**Fig 7:**
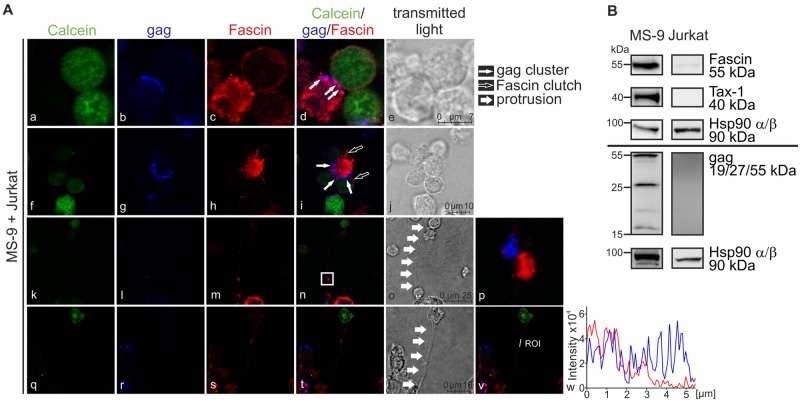
Fascin and gag localize at cell-cell contacts and in long-distance connections between infected and uninfected T-cells. **(A)** Confocal laser scanning microscopy of HTLV-1-infected MS-9 cells co-cultured with Jurkat T-cells. Jurkat T-cells were pre-stained with Calcein-AM (green) to differentiate between the two cell types. Cells were co-cultured for 0 (k-p), 30 (a-j) or 60min (q-w) on poly-L-lysine-coated coverslips prior to drying (20min), fixation, and staining. **(A)** Stainings of Calcein (green), gag (blue), Fascin (red) and the merge of all three stainings are shown. Transmitted light served as control. Representative stainings of three independent experiments showing clusters of Fascin (a-e) and gag (a-j), Fascin clutches (f-j) or long-distance connections (k-w) are depicted. Thin white arrows indicate gag of an infected cell clustering at the cell-cell contact towards an uninfected cell; framed white arrows indicate short-distance Fascin-containing membrane extensions; and thick white arrows indicate long-distance protrusions between uninfected and infected cells. Protrusions (k-o; q-u) were examined in more detail, and (p) the stains of gag and Fascin within the protrusion shown in (n) were enlarged; further, a region of interest (v-w) was analyzed showing the intensities of gag- (blue) and Fascin- specific (red) fluorescences shown in (t). **(B)** Detection of Fascin, Tax-1 and gag in HTLV-1-infected MS-9 cells and uninfected Jurkat T-cells by western blot. Hsp90 α/β served as control.

Having found that knockdown of Fascin impairs release and cell-to-cell transmission of HTLV-1, we performed imaging analysis to shed light on the localization of Fascin and of the viral gag protein (Fig 7A). Most chronically infected T-cell lines harbor more than one copy of HTLV-1 provirus and produce large amounts of gag, which is unfavorable for imaging analysis. Therefore, we decided to analyze the chronically infected T-cell line MS-9, which harbors only one integrated provirus and thus, reasonable amounts of gag to perform imaging analysis [26] (Fig 7B). MS-9 cells (Fascin-positive) were co-cultured with Jurkat T-cells that had been pre-stained with the live cell dye Calcein-AM (green) and express only low amounts of endogenous Fascin. Cells were spotted on poly-L-lysine- and fibronectin-coated coverslips and incubated for 0, 30, or 60min at 37°C before fixation. Afterwards, cells were stained with antibodies targeting Fascin (red) and gag p19 (blue), and confocal laser scanning microscopy was performed (Fig 7A). Imaging revealed different patterns of Fascin localization at cell-cell contacts. First, we examined cells where the viral gag protein polarizes towards the uninfected target cell, suggesting the presence of the virological synapse (VS) [21] (Fig 7Aa–7Aj). At cell-cell contacts we found not only clusters of gag (Fig 7Ab; thin white arrows), which are reminiscent of viral biofilms [24,53], but also clusters of Fascin protein (Fig 7Ac). Concomitant with our previous findings in MT-2 cells (Fig 6B; S7 Fig), we could rarely detect co-localizations between Fascin and gag. However, polarized gag clusters were interspersed with Fascin clusters (Fig 7Ad) at cell-cell contacts, which was confirmed by analysis of fluorescence intensities across the cell-cell-contact region (S8 Fig). This suggests that Fascin makes room for gag clusters at the VS. Since large aggregates of HTLV-1 virions in the viral biofilm on the surface of infected cells are important for efficient infection of target cells [53], Fascin could be important for the transport of viral proteins to the budding site, and thus, foster HTLV-1 transmission. This idea is supported by the quantitative evaluation of T-cell conjugates between MS-9 and Jurkat T-cells: If Fascin is localized at the cell-cell contact region, the frequency of polarized gag (suggesting formation of the VS) is 79.5% or 43.8% on poly-L-lysine or fibronectin, respectively. In contrast, if Fascin is dispersed and not accumulated at the cell-cell contact, the frequency of gag polarization (at the VS) is much lower (1.1% on poly-L-lysine, 1.8% on fibronectin). Thus, this suggests a direct role of Fascin in the local distribution of gag to budding sites and an indirect effect on cell-to-cell transmission. Beyond, we observed short, Fascin-containing membrane extensions that clutched uninfected T-cells (Fig 7Ai; white-framed arrows) and made room for these gag clusters (Fig 7Ai; thin white arrows). Finally, we found long-distance connections (approximately 15.21±5.48 μm in length on poly-L-lysine and 24.14±1.29μm on fibronectin) between infected MS-9 cells and uninfected Jurkat T-cells (Fig 7Ak–7Av; thick white arrows). The frequency of protrusions emanating from infected MS-9 cells was low (approximately 3.28% of all MS-9 cells), however, protrusions were found independent of the time point of analysis (Fig 7Ao and 7Au). Interestingly, we found Fascin and gag p19 protein expression in 65.3% or 79.5% of all protrusions (on poly-L-lysine or on fibronectin, respectively). Among the Fascin-positive protrusions all except one were also stained positive for gag p19. As depicted in Fig 7A, Fascin and gag partially co-localize in these cellular protrusions (Fig 7At, 7Av and 7Aw), or the proteins are located in clusters in close proximity (Fig 7An and 7Ap) between infected MS-9 cells and newly infected Jurkat T-cells. Thus, formation of Fascin-containing protrusions could potentially account for the transfer of gag to target cells.

**Fig 8:**
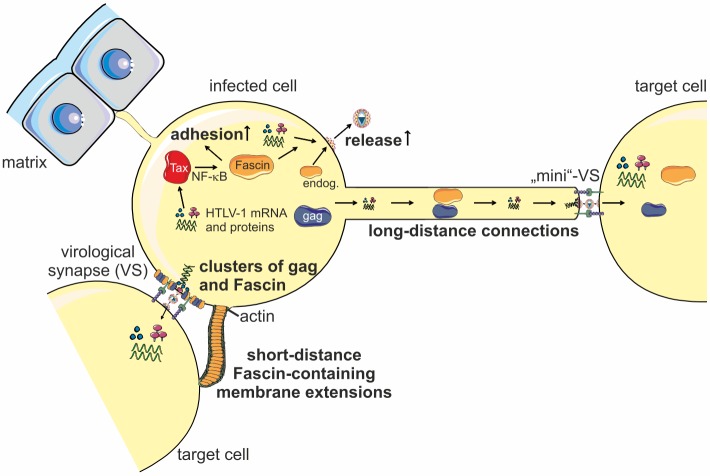
Model of Fascin’s role during HTLV-1 transmission. HTLV-1-infected T-cells express the transactivator Tax that upregulates Fascin expression via the NF-κB signaling pathway. Not only Tax-induced Fascin, but also endogenous (endog.) Fascin is required for virus release and cell-to-cell transmission. Beyond, adhesion of infected cells occurs Fascin-dependently, which may favor dissemination of infected cells *in vivo*. Functionally, Fascin makes room for gag clusters reminiscent of viral biofilms at the virological synapse (VS) and Fascin-containing short-distance membrane extensions clutch uninfected T-cells. Additionally, Fascin localizes with gag in long-distance connections between chronically infected and newly infected T-cells. A “mini VS” may be shaped at the tip of the long-distance connection towards the target cell. Overall, Fascin seems to be important for the transport of viral proteins to foster polarized budding, virus release and cell-to-cell transmission of HTLV-1.

To summarize our data, Fig 8 gives an overview of our current findings and provides a model of Fascin’s role in HTLV-1 transmission. HTLV-1-infected T-cells express the transactivator Tax that upregulates Fascin expression via the NF-κB signaling pathway. Not only Tax-induced Fascin, but also endogenous Fascin seems to be required for virus release and cell-to-cell transmission. Beyond, adhesion of infected cells in co-culture with uninfected cells occurs Fascin-dependently, which may favor dissemination of infected cells *in vivo*. Functionally, Fascin clusters intersperse with gag clusters suggesting that Fascin makes room for gag clusters reminiscent of viral biofilms at the VS. Furthermore, short-distance Fascin-containing membrane extensions clutch uninfected T-cells, which could favor the transfer of viral material to target cells via budding of enveloped virions at tight cell-cell contacts at the VS. Additionally, Fascin localizes with gag in long-distance connections between chronically infected and newly infected T-cells. It is conceivable that Fascin is required for the proper organization of protrusive structures, which may account for budding of HTLV-1 at the tip of the protrusion towards the target cell via a putative “mini VS”, a structure that had been proposed earlier [22,54]. Overall, our data suggest that Fascin could be important for the transport of viral proteins to foster polarized budding, virus release and cell-to-cell transmission of HTLV-1. Thus, Fascin is an interesting novel target to inhibit HTLV-1 cell-to-cell transmission.

## Discussion

In this work we found that the actin-bundling protein Fascin is critical for HTLV-1 transmission. Fascin is known as a tumor marker, which is highly upregulated in many types of cancer and crucial for invasion and metastasis, potentially by stabilizing invasive structures [30]. We previously showed that Fascin is also important for invasive migration of virus-transformed lymphocytes [29,42]. Using different cell culture systems and infection models, we now found that repression of Fascin by shRNA or by Fascin-specific nanobodies severely impairs release and cell-to-cell transmission of the retrovirus HTLV-1 shedding new light on the function of Fascin.

To address the role of Fascin in HTLV-1 cell-to-cell transmission, we made use of a single-cycle replication dependent reporter system, which allows automatic quantitation of productive infection in newly infected target cells only [25]. Using this system, our results indicate that Fascin is a major contributor of HTLV-1 cell-to-cell transmission independent of the cell type and the envelope type tested. Despite recent work showing that the reporter system we used even underestimates cell-to-cell transmission events [55], we still see a significant reduction of reporter gene activity reflecting cell-to-cell transmission upon repression of endogenous or Tax-induced Fascin expression. However, new reporter vectors with improved splicing and packaging of the spliced reporter RNA might allow for better quantitating the role of Fascin on cell-to-cell transmission in lymphocytes and in primary cells in future work [55].

Our data suggest that both virus release and cell-to-cell transmission appear Fascin-dependent, while the amount of cell-associated virus (as reflected by western blots of gag) is not affected by repression of Fascin. However, our data also suggest that Fascin’s role in cell-to-cell transmission dominates over its role on virus release. Despite a significant impact on cell-to-cell transmission, virus release was not affected by Fascin-specific shRNAs in every experimental condition (Fig 1C). This may be due to the fact that ELISAs measuring gag p19 also quantify non-infectious VLPs, while reporter assays (Figs 1–3) and flow cytometry measuring gag p19 transfer (Fig 4) quantitate newly-infected cells only. Although the release of virions is impaired upon knockdown of Fascin, cell-free virions carrying the wildtype env of HTLV-1 are severely impaired in infecting target cells (S3 Fig; [25]). Thus, it is very likely that reduced infectivity of co-cultured target cells upon knockdown of Fascin results from cell-to-cell transmission, and not from infection with poorly infectious free viral particles. Thus, the impact of Fascin on direct cell-to-cell transmission could be underestimated. To confirm the relevance of Fascin for HTLV-1 transmission, we also investigated chronically infected T-cells. Both fusion-based assays and measuring of gag transfer to target cells confirmed the relevance of Fascin for HTLV-1 release and cell-to-cell transmission.

Use of Fascin-specific nanobodies that target Fascin to the mitochondrial outer membrane (MOM) confirmed a role of Fascin in gag p19 release and HTLV-1 cell-to-cell transmission. Nanobodies are new promising stable recombinant antigen-binding domains of camelid heavy-chain antibodies that had already been successfully used to prevent Fascin-dependent invasion and migration of cancer cells [43,56]. Thus, nanobodies trigger Fascin protein loss of function without changing its expression [43]. Since the potent nanobody FASNb5 not only delocalizes Fascin to the MOM more efficiently, but also impairs Fascin-mediated organization of actin-bundles [43,56], Fascin’s actin-bundling activity might be required for transmission of HTLV-1. For HTLV-1, the role of the actin cytoskeleton on virus transmission has not been analyzed in detail, however, it is known that polarization of the MTOC and transfer of HTLV-1 reporter vectors to target cells is impaired in presence of compounds interfering with actin polymerization [21,25,57]. We found that not only repression of the actin-bundling protein Fascin, but also interference with actin and tubulin polymerization led to reduced gag p19 levels in the supernatants of HTLV-1-infected T-cells. These observations are in contrast to HIV, where the budding process does not strictly rely on cytoskeleton remodeling. Although filamentous actin co-localizes with budding structures, inhibition of actin does not change localization of budding sites and packaging of actin and actin-binding proteins into virions seems to be a secondary consequence of the high abundance of these molecules at budding sites [58].

Yet, it is unknown, whether Fascin also contributes to release and cell-to-cell transmission of other viruses than HTLV-1. We found that not only Tax [29], but also latent membrane protein 1 (LMP1) of Epstein-Barr virus (EBV) is a potent inducer of Fascin [42]. Interestingly, LMP1-deleted EBV is severely impaired in virus release into culture supernatants, potentially due to a defect in particle transport [59]. Thus, LMP1-mediated induction of Fascin and its continuous expression suggest a role of Fascin in virus release also of EBV. This is further corroborated by the finding that cell-to-cell transmission of EBV to epithelial cells also depends on canonical NF-κB signaling [60], which is a prerequisite for efficient Fascin induction by LMP1 [42].

Although it is known that Tax is required for formation of the VS and efficient virus transmission [21], only little is known about host factors that are regulated by Tax to modulate virus transmission [17]. With regard to pathways important for viral transmission, Tax transcriptionally alters the expression of cell adhesion and surface molecules, leads to cytoskeletal remodeling and complexes with proteins involved in cytoskeleton structure and dynamics. These Tax-interacting proteins include α-internexin, cytokeratin, actin, gelsolin, annexin, γ-tubulin and small GTPases of the Rho family [61] Two of these Rho-GTPases, Rac-1 and Cdc42, complex with Tax and seem to be important for Tax-induced MTOC-polarization [57,62]. Thus, it is conceivable that Tax might connect Rho GTPases to their targets and affect cytoskeleton organization which could favor HTLV-1 transmission. Interestingly, Chevalier *et al*. found that GEM, which is an upstream negative regulator of ROCK-I Rho kinase, is induced by Tax [28]. GEM is a small GTP-binding protein and enhances cellular migration and conjugate formation between infected and uninfected T-cells. Knockdown of GEM in chronically infected T-cells reduces gag transfer to target cells showing that GEM is required for viral transmission [28]. It had been suggested earlier that not only GEM, but also Fascin and collapsin response mediator protein 2 (CRMP2), which is induced by Tax and important for migration [63], might contribute to HTLV-1 transmission [17,28]. We now show that this is true for Fascin, however, the mechanism differs from the one described for GEM. Both GEM and Fascin are important for HTLV-1 cell-to-cell transmission, whereas only GEM is required for T-cell conjugate formation between infected and uninfected T-cells [28]. Contrary, Fascin also impairs virus release, which seems to be unaffected by GEM.

Our findings that the adhesion of infected cells to different matrices is modulated by Fascin, in co-cultures with uninfected cells, could explain our previous observations where we found Fascin to be important for the invasion of ATL-derived cells through ECM and for the invasive migration of EBV-transformed and LMP-1-expressing lymphocytes [29,42]. These results are in line with recent data showing that Fascin forms a complex with focal adhesion kinase (FAK) and Src to control adhesion stability [31]

Contrary to the test systems used in our manuscript, free viral particles of HTLV-1 are hardly detectable *in vivo* [20]. Viruses are transmitted at tight cell-cell contacts or via cellular protrusions protected from the host’s immune response [21,22]. It is estimated that HTLV-1 buds into a synaptic cleft and is transferred to target cells [21]. Moreover, viruses are tethered to and embedded in extracellular assemblies, viral biofilms, and transmitted at virological synapses to target cells [24]. It is likely that immune pressure and specific signals from uninfected target cells play a role in preventing release of HTLV-1 *in vivo*. Thus, it remains to be determined how Fascin affects HTLV-1 transmission in natural infection.

Imaging revealed that Fascin clusters localize in close proximity to gag clusters at cell-cell-contacts, which are reminiscent of viral biofilms. Viral biofilms are carbohydrate-rich surface assemblies of viral particles which are composed of various components of the ECM and they account for the majority of HTLV-1 cell-to-cell transmission *in vitro* [24]. The localization of Fascin in close proximity to gag suggests that Fascin makes room for gag clusters at viral biofilms. Beyond, it is also conceivable that Fascin is required for formation, maintenance or tethering of viral biofilms, e.g. by redirecting the transport of viral and cellular proteins to budding sites via reorganization of the actin- or microtubuli-cytoskeleton [30,31]. Since Fascin is concentrated at cell-cell contacts, and localizes in close proximity to gag clusters, it is possible that Fascin may be packaged into HTLV-1 particles. We also observed short, Fascin-containing short membrane extensions clutching uninfected T-cells. These potentially support the transfer of virions to target cells, but presumably not due to enhanced conjugate formation, which remains unaffected by Fascin. Surprisingly, we observed Fascin and gag localization in long-distance protrusions between chronically infected and newly-infected T-cells. Long distance connections for the transfer of retroviruses or viral proteins have previously also been found in cells infected with MLV [64] or HIV [65]. For HTLV-1, the viral p8 protein was identified as inducer of cellular protrusions [22]. Therefore, it remains to be determined, whether p8-induced protrusions are Fascin-dependent, and whether viruses bud from these protrusions at a “mini VS” to target cells.

Taken together, our data suggest that Fascin could be important for the transport of viral proteins to budding sites, and thus, foster HTLV-1 transmission. However, the detailed mechanism of Fascin-dependent HTLV-1 transmission remains to be determined. Since repression of Fascin also reduces release of gag p19 into culture supernatants, it is conceivable that either the transport of viral proteins to the budding sites is impaired, or that viral particles are retained inside the infected cell, or at the viral biofilms. Since co-localization events between Fascin and gag were rare, our findings suggest a transient or indirect Fascin:gag interaction during the dynamic process of virus budding. Despite playing a crucial role in cell-to-cell transmission of HTLV-1, it is not settled yet whether Fascin is also essential for formation of the VS. Localization of Fascin at cell-cell contacts and its association with a high frequency of polarized gag suggests that Fascin is involved in recruiting gag to the VS, and, thus, indirectly affects cell-to-cell transmission. However, it is unclear whether gag protein could localize at the VS in the absence of Fascin. These experiments are not accomplishable with chronically infected MT-2 cells, which can be manipulated by knockdown strategies, since these cells carry several proviral copies- some of them defective [66]- and excessive amounts of cell-associated gag protein.

Fascin may also represent an interesting regulator of HTLV-1 cell-to-cell transfer in other cell types than infected T-cells. It is estimated that dendritic cells (DC) are the primary cells to be infected *in vivo* and that they play a pivotal role in transmitting the virus to CD4^+^ T-cells depending on cell-cell-contacts. Beyond, infection of DC may also be required for the establishment and maintenance of HTLV-1 infection in primate species [67]. Contrary to CD4^+^ T-cells, DCs are efficiently infected cell-free by highly concentrated viruses or by separated viral biofilms *in vitro* [68,69]. Since Fascin expression is selectively induced in mature DC [70], important for the stability of dendrites and for formation of the immunological synapse [32], future work should also investigate whether Fascin plays a role in dissemination of HTLV-1 from DC to T-cells.

For a long time, Fascin has been known as an actin-bundling protein only. However, Fascin exerts other functions independent of its role in actin-binding and -bundling. Recent findings have supported this notion showing that Fascin also interacts with microtubules [31]. In light of HTLV-1 transmission, which depends on polarization of the MTOC and on proper actin and tubulin function [21,25], our work identifying Fascin as a critical host factor in HTLV-1 transmission may provide a link between the activity of Tax and regulation of both the actin and microtubule cytoskeleton. Thus Fascin is a promising candidate to counteract HTLV-1 transmission.

## Supporting Information

S1 Fig Steps of automatic image analysis of immunofluorescence stainings of MT-2 cells in co-culture with Jurkat T-cells. **(a)** Detection of Jurkat T-cells using Hough-voting. **(b)** Segmentation of adjacent and overlapping Jurkat T-cells using active contours. **(c)** Masking Fascin and gag-negative Jurkat T-cells in AlexaFluor350 channel (gag p19-staining of MT-2). **(d)** Detection of MT-2 cells using Hough-voting. **(e)** Segmentation of adjacent and overlapping stained MT-2 cells using active contours and active shape models.
(TIF)

S2 Fig Fascin knockdown by specific shRNAs does not affect cell vitality of 293T cells. **(A-B)** 293T cells were transfected with shRNA constructs carrying an IRES-EGFP expression cassette and shRNAs targeting Fascin (shFascin5, shFascin4) or a control (shNonsense), and cells were selected with puromycin (4μg/ml) for 6 days. **(A)** Flow cytometry monitoring GFP expression. **(B)** Live/dead staining using AnnexinV/Sytox-AADvanced. The means of four independent experiments ± standard error (SE) are shown.
(TIF)

S3 Fig Comparison of cell-free and cell-to-cell transmission levels using the single-cycle replication-dependent reporter system in a co-culture of Jurkat T-cells with Raji/CD4+ B-cells. Jurkat T-cells were transfected with the reporter vector pCRU5HT1M-inluc (inluc) and the packaging plasmid pCMVHT1M encoding HTLV-1 with wildtype env (wt). Cells were co-transfected with pEF (mock) and a shRNA control (shNonsense). After 24h, Raji/CD4^+^ B-cells were either incubated with the supernatants of the transfected Jurkat T-cells (cell-free transmission) or co-cultured with the transfected Jurkat T-cells (cell-to-cell transmission). Luciferase assays were performed after 48h to compare cell-free with cell-to-cell transmission levels. The means of four independent experiments ± standard error (SE) are shown and relative light units (RLUs) were compared using Student’s t-tests (**: p<0.01).
(TIF)

S4 Fig qPCR analysis of *Tax* and *Fascin* transcripts in Jurkat T-cells and 293T cells. **(A-B)** Jurkat T-cells (left) and 293T cells (right) were transfected with pEFTax or pEF (mock) 48h prior to qPCR experiments. **(A)** qPCR analysis depicting the relative copy numbers (rcn) of *Fascin* transcripts normalized on *β-actin* (*ACTB*). The means of three independent experiments ± standard error (SE) are shown and compared to pEF using Student’s t-test (**: p<0.01). **(B)** Detection of Fascin and Tax-1 in Jurkat T-cells (left) and 293T cells (right) by western blot. β-actin (ACTB) served as control.
(TIF)

S5 Fig Fascin knockdown by specific shRNAs does not affect cell vitality of chronically HTLV-1-infected T-cells. Staining of HuT-102 and MT-2 cells using AnnexinV and Live/Dead. Additionally, MT-2 cells were either left untreated or treated with DMSO (control) or 15μM etoposide (apoptosis control). Number (%) of HuT-102 and MT-2 cells that are either positive for AnnexinV, Live/Dead or both stainings. The means of three independent experiments ± standard error (SE) are shown.
(TIF)

S6 Fig Evaluation strategy of cell-cell aggregation experiments with Jurkat T-cells co-cultured with Raji/CD4^+^ B-cells using flow cytometry. **(A-B)** Transfection, co-culture and stainings were performed as described in Fig 5 (see Cell-cell aggregation assay in Materials and Methods). **(A)** GFP-positive (green) and -negative cells (blue) of Raji/CD4^+^ B-cells (RC) co-cultured with transfected Jurkat T-cells (J). Cells transfected with pEFTax and shNonsense (shNon) are shown as representative dot plots. **(B)** CD3-AlexaFluor700 (CD3-AF700; Jurkat) and HLA-DR-PacificBlue (Raji/CD4^+^) stainings of GFP-positive and GFP-negative cells of **(A)**. Double stainings indicate aggregation formation of Jurkat T-cells with Raji/CD4^+^ B-cells.
(TIF)

S7 Fig Localization of Fascin and gag. Immunofluorescence stainings of co-cultures between MT-2 cells and Jurkat T-cells (1h, 37°C, on poly-L-lysine-coated coverslips). Jurkat cells were pre-stained with Calcein-AM (green, a) to differentiate between the two cell types. Stainings of gag (blue, b), Fascin (red, c) and the merge of all three stainings (d) are shown. Arbitrary regions of interest (ROIs) were drawn, and fluorescence intensities of gag- and Fascin-specific stains were quantitatively evaluated along the ROIs (f, g).
(TIF)

S8 Fig Localization of Fascin and gag at the virological synapse. Confocal laser scanning microscopy of HTLV-1-infected MS-9 cells co-cultured with Jurkat T-cells as shown in Fig 7Aa–7Ad. Jurkat T-cells were pre-stained with Calcein-AM (green) and co-cultured for 30 min on poly-L-lysine-coated coverslips prior to drying (20min), fixation, and staining. Stainings of Calcein (green, a), gag (blue, b), Fascin (red, c) and the merge of all three stainings (d) are shown. A region of interest (ROI) was drawn across the cell-cell-contact region (e) reflecting the virological synapse, and fluorescence intensities of gag- and Fascin-specific stains were quantitatively evaluated along the ROIs (f).
(TIF)
