# Supplementary figures and images for: The Tax-Inducible Actin-Bundling Protein Fascin Is Crucial for Release and Cell-to-Cell Transmission of Human T-Cell Leukemia Virus Type 1 (HTLV-1)
Source: PLoS Pathog. 2016 Oct 24;12(10):e1005916. doi: 10.1371/journal.ppat.1005916 (PMC5077169; doi:10.1371/journal.ppat.1005916)

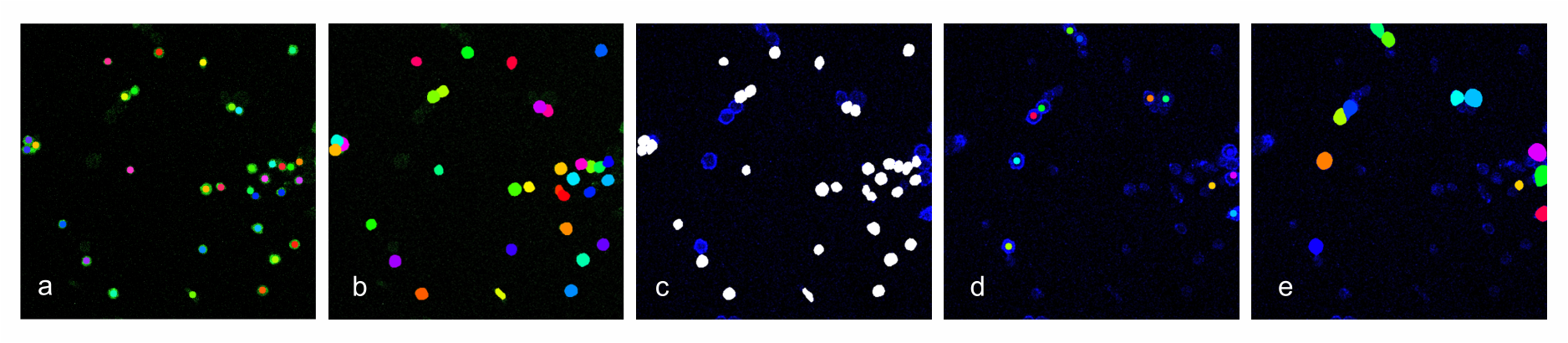

Supplement: S1 Fig — (a) Detection of Jurkat T-cells using Hough-voting. (b) Segmentation of adjacent and overlapping Jurkat T-cells using active contours. (c) Masking Fascin and gag-negative Jurkat T-cells in AlexaFluor350 channel (gag p19-staining of MT-2). (d) Detection of MT-2 cells using Hough-voting. (e) Segmentation of adjacent and overlapping stained MT-2 cells using active contours and active shape models. (TIF) [file ppat.1005916.s001.tif]

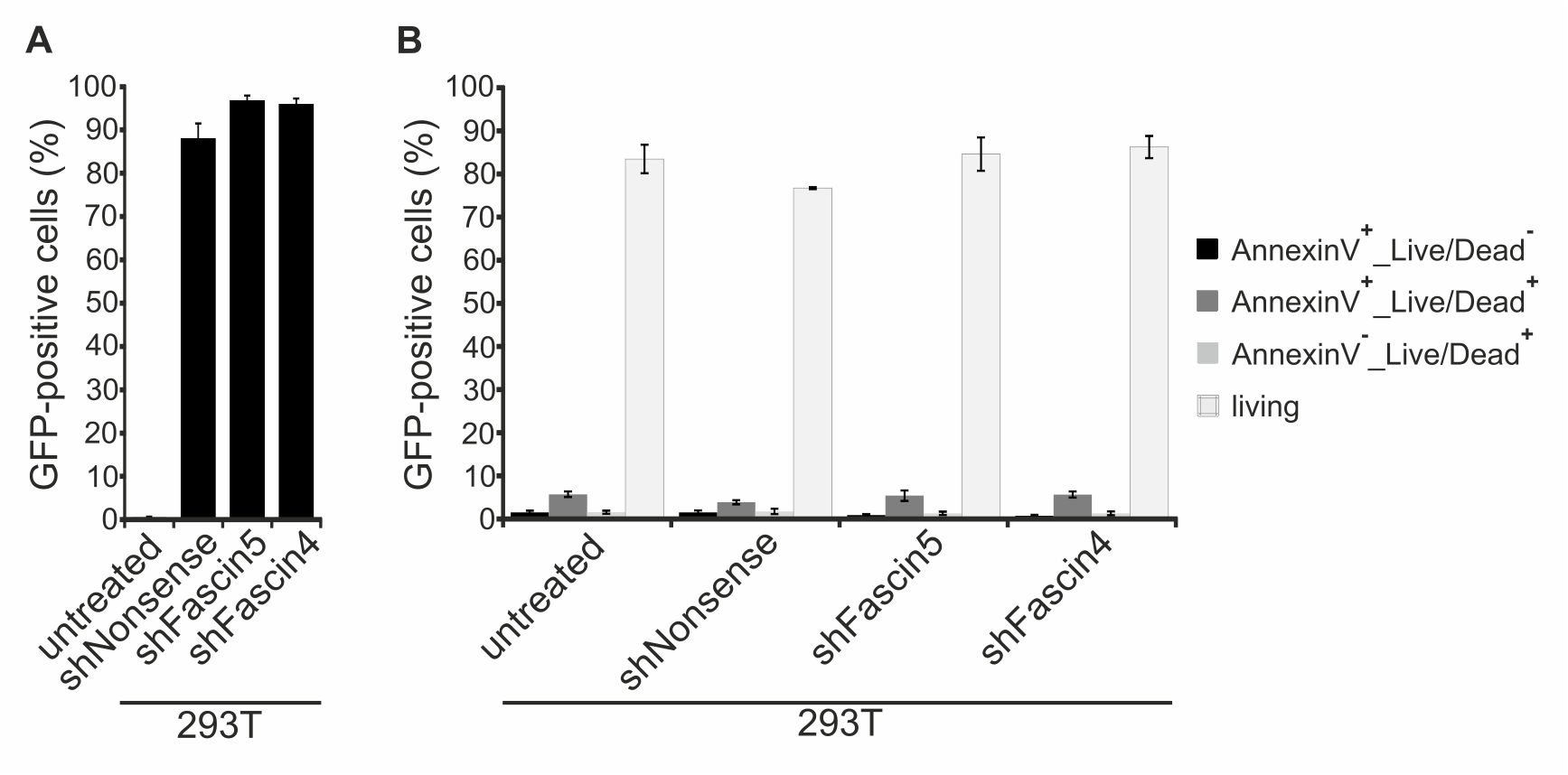

Supplement: S2 Fig — (A-B) 293T cells were transfected with shRNA constructs carrying an IRES-EGFP expression cassette and shRNAs targeting Fascin (shFascin5, shFascin4) or a control (shNonsense), and cells were selected with puromycin (4μg/ml) for 6 days. (A) Flow cytometry monitoring GFP expression. (B) Live/dead staining using AnnexinV/Sytox-AADvanced. The means of four independent experiments ± standard error (SE) are shown. (TIF) [file ppat.1005916.s002.tif]

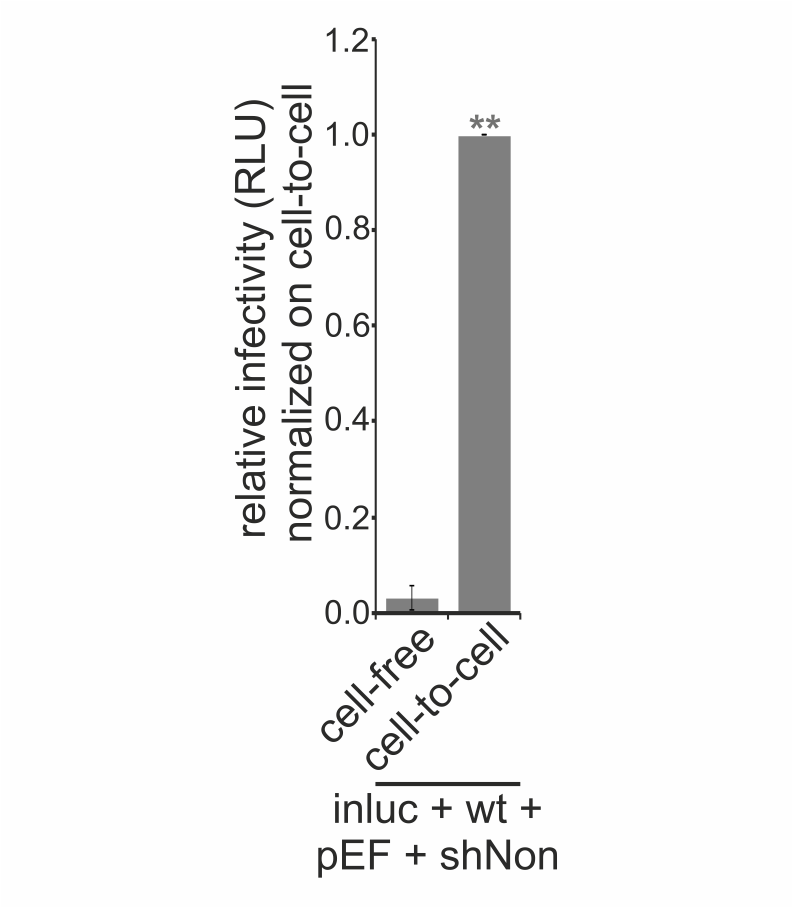

Supplement: S3 Fig — Jurkat T-cells were transfected with the reporter vector pCRU5HT1M-inluc (inluc) and the packaging plasmid pCMVHT1M encoding HTLV-1 with wildtype env (wt). Cells were co-transfected with pEF (mock) and a shRNA control (shNonsense). After 24h, Raji/CD4+ B-cells were either incubated with the supernatants of the transfected Jurkat T-cells (cell-free transmission) or co-cultured with the transfected Jurkat T-cells (cell-to-cell transmission). Luciferase assays were performed after 48h to compare cell-free with cell-to-cell transmission levels. The means of four independent experiments ± standard error (SE) are shown and relative light units (RLUs) were compared using Student’s t-tests (**: p<0.01). (TIF) [file ppat.1005916.s003.tif]

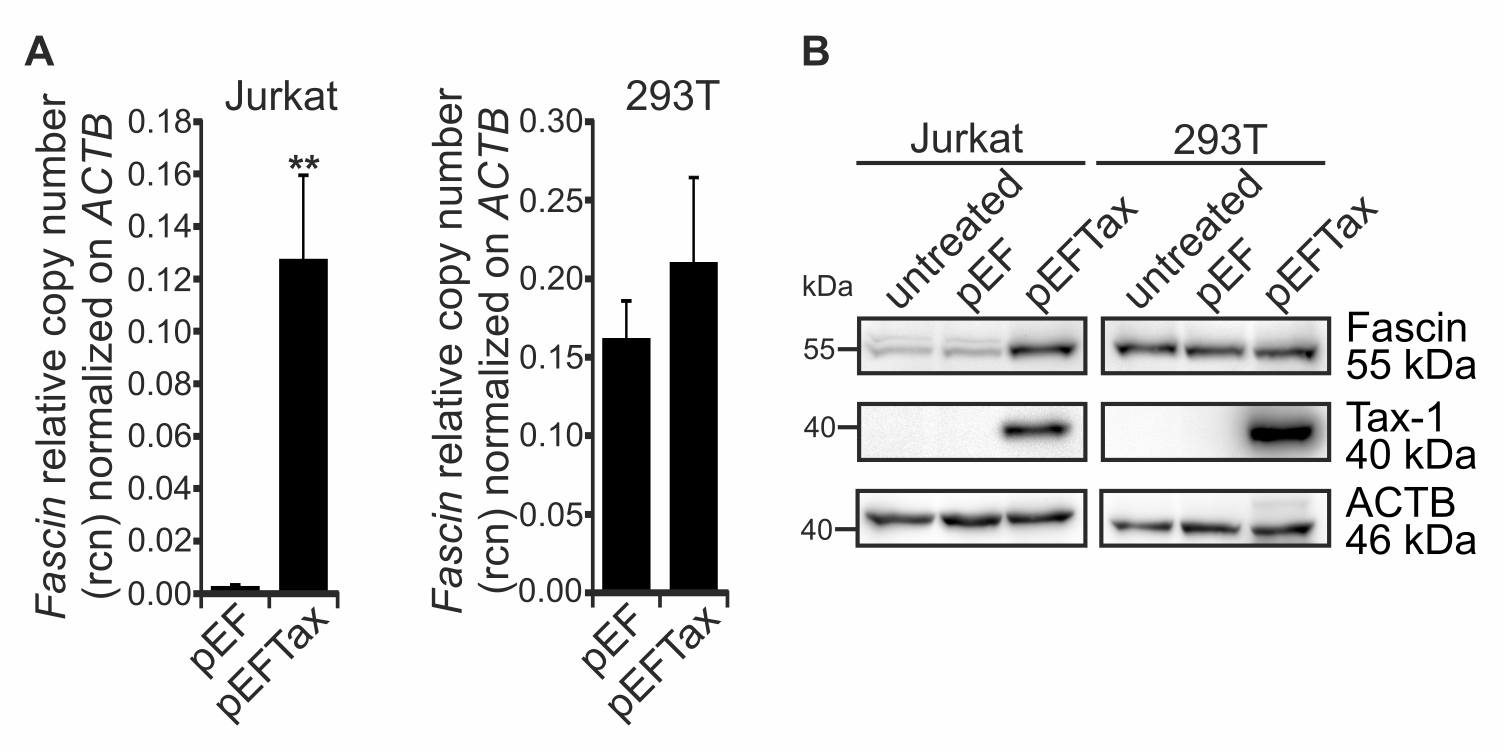

Supplement: S4 Fig — (A-B) Jurkat T-cells (left) and 293T cells (right) were transfected with pEFTax or pEF (mock) 48h prior to qPCR experiments. (A) qPCR analysis depicting the relative copy numbers (rcn) of Fascin transcripts normalized on β-actin (ACTB). The means of three independent experiments ± standard error (SE) are shown and compared to pEF using Student’s t-test (**: p<0.01). (B) Detection of Fascin and Tax-1 in Jurkat T-cells (left) and 293T cells (right) by western blot. β-actin (ACTB) served as control. (TIF) [file ppat.1005916.s004.tif]

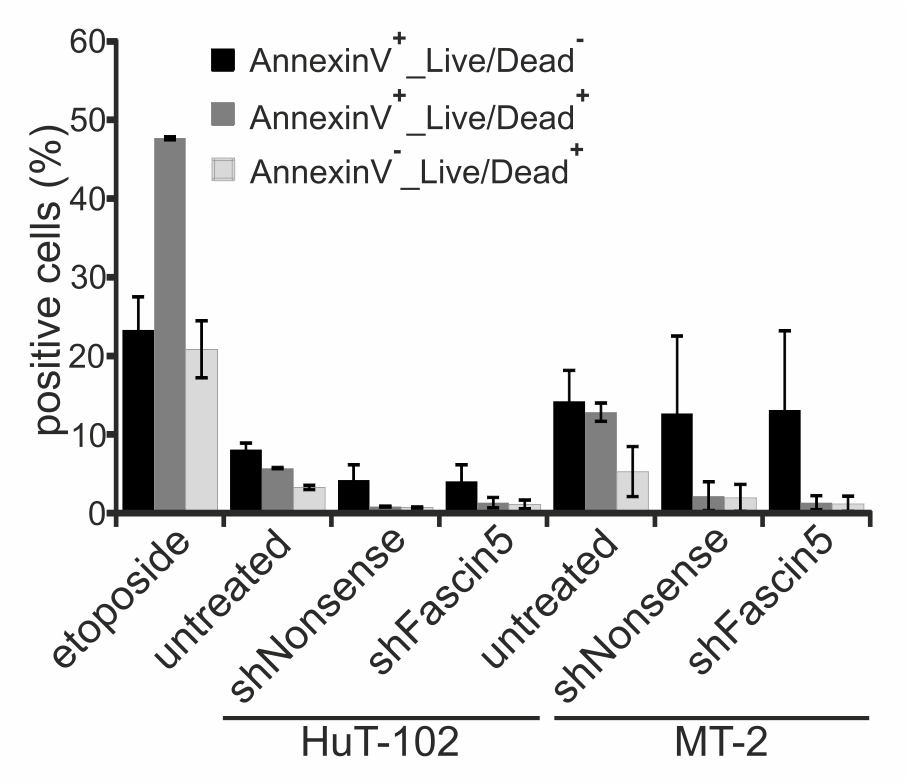

Supplement: S5 Fig — Staining of HuT-102 and MT-2 cells using AnnexinV and Live/Dead. Additionally, MT-2 cells were either left untreated or treated with DMSO (control) or 15μM etoposide (apoptosis control). Number (%) of HuT-102 and MT-2 cells that are either positive for AnnexinV, Live/Dead or both stainings. The means of three independent experiments ± standard error (SE) are shown. (TIF) [file ppat.1005916.s005.tif]

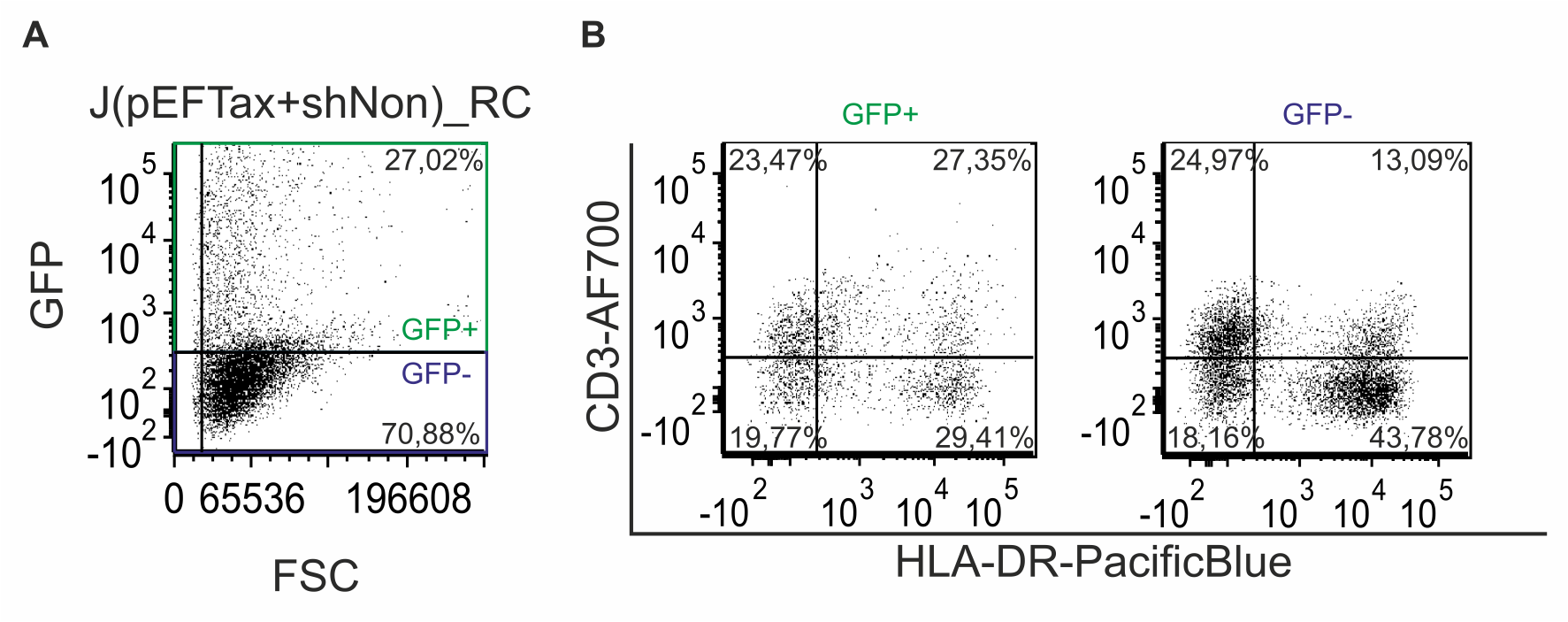

Supplement: S6 Fig — (A-B) Transfection, co-culture and stainings were performed as described in Fig 5 (see Cell-cell aggregation assay in Materials and Methods). (A) GFP-positive (green) and -negative cells (blue) of Raji/CD4+ B-cells (RC) co-cultured with transfected Jurkat T-cells (J). Cells transfected with pEFTax and shNonsense (shNon) are shown as representative dot plots. (B) CD3-AlexaFluor700 (CD3-AF700; Jurkat) and HLA-DR-PacificBlue (Raji/CD4+) stainings of GFP-positive and GFP-negative cells of (A). Double stainings indicate aggregation formation of Jurkat T-cells with Raji/CD4+ B-cells. (TIF) [file ppat.1005916.s006.tif]

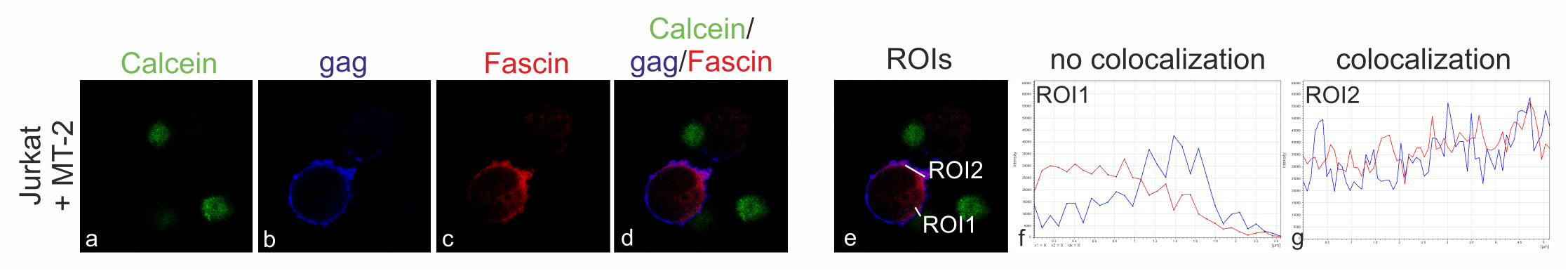

Supplement: S7 Fig — Immunofluorescence stainings of co-cultures between MT-2 cells and Jurkat T-cells (1h, 37°C, on poly-L-lysine-coated coverslips). Jurkat cells were pre-stained with Calcein-AM (green, a) to differentiate between the two cell types. Stainings of gag (blue, b), Fascin (red, c) and the merge of all three stainings (d) are shown. Arbitrary regions of interest (ROIs) were drawn, and fluorescence intensities of gag- and Fascin-specific stains were quantitatively evaluated along the ROIs (f, g). (TIF) [file ppat.1005916.s007.tif]

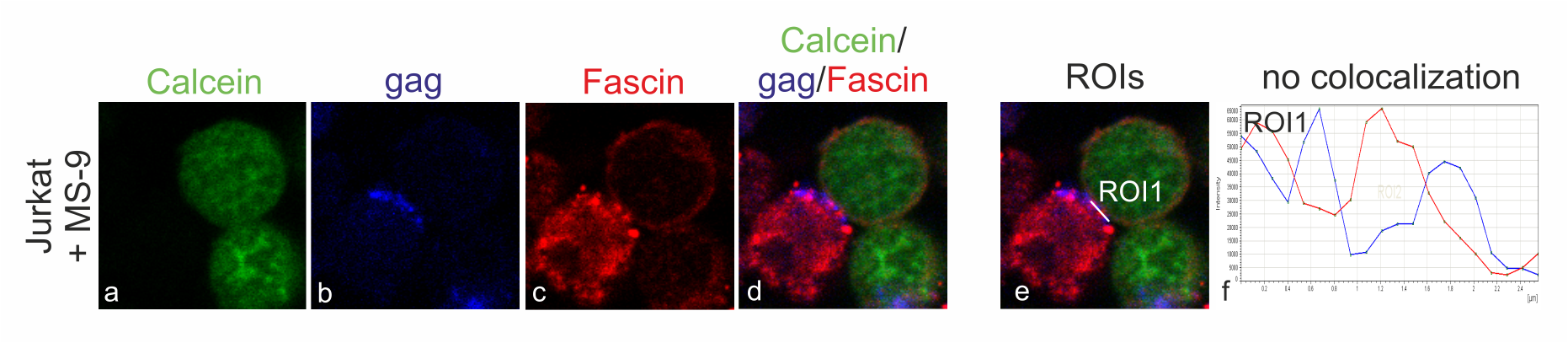

Supplement: S8 Fig — Confocal laser scanning microscopy of HTLV-1-infected MS-9 cells co-cultured with Jurkat T-cells as shown in Fig 7Aa–7Ad. Jurkat T-cells were pre-stained with Calcein-AM (green) and co-cultured for 30 min on poly-L-lysine-coated coverslips prior to drying (20min), fixation, and staining. Stainings of Calcein (green, a), gag (blue, b), Fascin (red, c) and the merge of all three stainings (d) are shown. A region of interest (ROI) was drawn across the cell-cell-contact region (e) reflecting the virological synapse, and fluorescence intensities of gag- and Fascin-specific stains were quantitatively evaluated along the ROIs (f). (TIF) [file ppat.1005916.s008.tif]
